# AGeNNT: annotation of enzyme families by means of refined neighborhood networks

**DOI:** 10.1186/s12859-017-1689-6

**Published:** 2017-05-25

**Authors:** Florian Kandlinger, Maximilian G. Plach, Rainer Merkl

**Affiliations:** 1 Institute of Biophysics and Physical Biochemistry, University of Regensburg, D-93040 Regensburg, Germany; 2 Faculty of Mathematics and Computer Science, University of Hagen, D-58084 Hagen, Germany

**Keywords:** Sequence similarity network, SSN, Genome neighborhood network, GNN, Genome content, Enzyme function, Homology-free annotation

## Abstract

**Background:**

Large enzyme families may contain functionally diverse members that give rise to clusters in a sequence similarity network (SSN). In prokaryotes, the genome neighborhood of a gene-product is indicative of its function and thus, a genome neighborhood network (GNN) deduced for an SSN provides strong clues to the specific function of enzymes constituting the different clusters. The Enzyme Function Initiative (http://enzymefunction.org/) offers services that compute SSNs and GNNs.

**Results:**

We have implemented AGeNNT that utilizes these services, albeit with datasets purged with respect to unspecific protein functions and overrepresented species. AGeNNT generates refined GNNs (rGNNs) that consist of cluster-nodes representing the sequences under study and Pfam-nodes representing enzyme functions encoded in the respective neighborhoods. For cluster-nodes, AGeNNT summarizes the phylogenetic relationships of the contributing species and a statistic indicates how unique nodes and GNs are within this rGNN. Pfam-nodes are annotated with additional features like GO terms describing protein function. For edges, the coverage is given, which is the relative number of neighborhoods containing the considered enzyme function (Pfam-node). AGeNNT is available at https://github.com/kandlinf/agennt.

**Conclusions:**

An rGNN is easier to interpret than a conventional GNN, which commonly contains proteins without enzymatic function and overly specific neighborhoods due to phylogenetic bias. The implemented filter routines and the statistic allow the user to identify those neighborhoods that are most indicative of a specific metabolic capacity. Thus, AGeNNT facilitates to distinguish and annotate functionally different members of enzyme families.

**Electronic supplementary material:**

The online version of this article (doi:10.1186/s12859-017-1689-6) contains supplementary material, which is available to authorized users.

## Background

A common method for annotating a protein is homology-based transfer of function by means of sequence comparison. Possible matches are organized in databases like InterPro [1] or Pfam [2] and the usage of such databases simplifies the assignment of protein function due to the comprehensive characterization of their entries. InterPro comprises signatures from more than ten repositories. Pfam entries subsume sequences and functions of individual protein domains which are accessed by their Pfam-ID.

However, the level of misannotation in some databases can exceed 80%, if sequence similarity is the only measure to assign function [3]. Reliability increases with the integration of orthogonal methods like genome neighborhoods (GNs). In prokaryotic genomes, genes are organized in operons and commonly, the corresponding gene-products have related functions like the enzymes that catalyze subsequent steps of a metabolic pathway. Thus, GN algorithms utilize the fact that short distances between genes allow for the prediction of a functional coupling of their products, if the GN is conserved across many phylogenetically diverse species [4]. Such GNs are particularly useful to characterize elements of large enzyme superfamilies. More than a third of them are functionally diverse, *i. e.*, homologous members catalyze reactions with different EC numbers [5]. If these homologs are part of different operons, the GNs of isofunctional enzymes must be similar and a GN comparison must discriminate functionally different enzymes.

To identify putatively isofunctional enzymes, one can compare their sequences pairwise and cluster them, if sequence similarity exceeds a superfamily-specific threshold *Th*. However, depending on the *Th* value, clusters may break or regroup. Thus, sequence similarity networks (SSNs) that represent sequences as nodes and their pairwise similarity as weighted edges are a more flexible concept to model subtle sequence relationships that may interlink sequence clusters [6]. It follows that the set of GNs deduced for all nodes of an SSN also form networks, commonly named genome neighborhood networks (GNNs).

In order to facilitate the prediction of specific enzyme functions, the Enzyme Function Initiative (EFI, http://enzymefunction.org/) is developing and disseminating high throughput *in silico* methods and offers services that compute for a given set of protein sequences SSNs and GNNs. In this context, the GN of a given gene product is represented by a set of Pfam-IDs, listing all protein domains found in the adjacent ±*nb* neighbors, where *nb* is chosen by the user. To date, these are the only GNNs deduced from a large number of genomes. We have implemented AGeNNT that Automatically Generates refined Neighborhood NeTworks. AGeNNT utilizes the EFI services but processes in- and output in order to create function-oriented, intuitive and easy to interpret GNNs.

## Methods

### Computing the thresholds *A-Th* and *S-Th*

The two alternative thresholds *A-Th* and *S-Th* computed by AGeNNT are based on the analysis of two values that result if a chosen threshold (*Th*) is used to eliminate edges of an SSN: According to terminology introduced earlier [7], *Nn*(*Th*) is the resulting number of nodes interconnected by edges and *SE*(*Th*) is the resulting number of edges. Both network parameters can be combined to a ratio value *Nsv*(*Th*):

1 $$ N s v(Th)=\frac{SE(Th)}{Nn(Th)} $$

Applying a low *Th*-value induces the predominant elimination of edges that connect nodes belonging to *different* protein families, because their pairwise sequence similarity is low. Consequently, the increase of *Th* decreases *SE*(*Th*) but not *Nn*(*Th*), as these nodes are still connected to other members of the same family. Thus, the ratio *Nsv*(*Th*) becomes smaller until the specific *Th* value is reached that also induces the elimination of edges within sequence clusters. For this *Th* value and larger ones, isolated nodes will arise that increase *Nsv*(*Th*). By step-wise incrementing *Th*_*t*_ to *Th*_*t*+1_, AGeNNT determines the lowest threshold inducing the raise of *Nsv*(*Th*); this is the *A-Th* value computed according to [7].

We suggest an alternative, smoothed threshold named *S-Th* that is based on the relative changes *relNn*(*Th*) and *relSE*(*Th*):

2 $$ relNn\left( T h\right)=\frac{Nn\left( T h\right)}{Nn\left( T{h}_{min}\right)}, relSE\left( T h\right)=\frac{SE\left( T h\right)}{SE\left( T{h}_{min}\right)} $$

Here, *Th*_*min*_ is the smallest pairwise similarity score occurring within the considered SSN. We define *S-Th* as the lowest value *Th*_*t*_ for which the gain of isolated nodes is higher than the loss of eliminated edges:

3 $$ S\hbox{-} T h=\underset{T{h}_t}{\mathrm{argmin}}\left(\left( relNn\left( T{h}_t\right)- relNn\left( T{h}_{t+1}\right)\right)>\left( relSE\left( T{h}_t\right)- relSE\left( T{h}_{t+1}\right)\right)\right) $$

### Computing a measure for the uniqueness of Pfam-nodes and cluster-nodes

Inspired by the work of A. Kalinka [8], we utilized the hypergeometric distribution in order to assess the uniqueness of network elements. A GNN is a graph consisting of cluster-node *c*_*j*_ (representing *n* sequence-nodes *s*_*i*_ from the corresponding SSN), Pfam-nodes *p*_*l*_, and edges *e*_*i*_^*l*^ = (*s*_*i*_, *p*_*l*_) interconnecting a sequence-node and a Pfam-node.

For a given GNN, let *N* = |{*s*_*i*_}| be the total number of sequence-nodes. Let *M*_*l*_ = |{*e*_*i*_^*l*^}| be the number of sequence-nodes interconnected to a specific *p*_*l*_ and let *k*_*l*_^*j*^ = |{*s*_*i*_ ∈ *c*_*j*_| ∃ *e*_*i*_^*l*^}| be the number of sequence-nodes *s*_*i*_ belonging to *c*_*j*_ and connected to a certain *p*_*l*_. Using the hypergeometric distribution, one can compute the probability that *k*_*l*_^*j*^ out of the *n* sequence-nodes have *p*_*l*_ in their GN:

4 $$ {P}_{p_l}^{c_j}\left( X={k}_l^j\right)=\frac{\left(\begin{array}{c}\hfill {M}_l\hfill \\ {}\hfill {k}_l^j\hfill \end{array}\right)\left(\begin{array}{c}\hfill N-{M}_l\hfill \\ {}\hfill n-{k}_l^j\hfill \end{array}\right)}{\left(\begin{array}{c}\hfill N\hfill \\ {}\hfill n\hfill \end{array}\right)} $$

As a measure of the “uniqueness” of an edge (cluster-node *c*_*j*_, Pfam-node *p*_*l*_), AGeNNT lists the following value:

5 $$ unique\left({p}_l,{c}_j\right)=-{ \log}_{10}\left({P}_{p_l}^{c_j}\left({k}_l^{{}_j}\right)\right) $$

As an approximation, we assume independence of the occurrence of the different Pfam-nodes *p*_*l*_ that belong to the GN of cluster-node *c*_*j*_. Thus, we determine the uniqueness of a GN for a certain cluster *c*_*j*_ according to:

6 $$ unique\left({c}_j\right)={\displaystyle \sum_{p_l\in pfam\left({c}_j\right)}-{ \log}_{10}\left({P}_{p_l}^{c_j}\left({k}_l^j\right)\right)} $$

Here, *pfam*(*c*_*j*_) is the set of Pfam-nodes interconnected to sequence-nodes belonging to *c*_*j*_. A high uniqueness value of a cluster-node arises, if its GN consists of many, exclusively linked Pfam-nodes. Analogously, AGeNNT computes the uniqueness of Pfam-nodes:

7 $$ unique\left({p}_l\right)={\displaystyle \sum_{c_j\in cluster\left({p}_l\right)}-{ \log}_{10}\left({P}_{p_l}^{c_j}\left({k}_l^j\right)\right)} $$

Here, *cluster*(*p*_*l*_) is the set of cluster-nodes interconnected to the Pfam-node *p*_*l*_.

### Eliminating subspecies

In SSNs deduced from InterPro families, sequences are annotated with a unique number (taxid) indicating their phylogenetic origin. Thus, for these well characterized datasets, AGeNNT can correct for strongly overrepresented species. To do so, AGeNNT utilizes a list (ftp://ftp.ncbi.nlm.nih.gov/pub/taxonomy/taxcat.zip) generated by the NCBI that indicates for each taxid whether it belongs to a species or a subspecies. At wish, AGeNNT eliminates all sequences originating from the genomes of subspecies prior to the computation and analysis of an rGNN.

### User-defined whitelist

The user can specify a whitelist consisting of a text file containing one Pfam-ID per line. Pfams that are not part of this whitelist will be skipped during the process of generating a filtered GNN. The built-in whitelist consists of 7176 Pfams-IDs whose description contains a reference to an enzymatic function like an EC number or the terms “enzyme” or “catalytic”.

### Listing the phylogenetic origin of sequences

Based on taxids, AGeNNT determines for each cluster-node the normalized frequencies with which the different phyla contribute sequences. These numbers are listed as *PhylumStat* values.

### Download and installation of AGeNNT

AGeNNT is a standalone Java application that can be used on many computer platforms. However, for the analysis of large networks (especially SSNs), we recommend a powerful CPU and at least 32 GB main memory. After download, AGeNNT can be started without any additional configuration by executing a command script. See the README file to be found after installation in the ..\agennt directory. For the generation of neighborhood networks and their visualization, the program Cytoscape is needed. It can be downloaded from http://www.cytoscape.org/. During the first call of Cytoscape within AGeNNT, the user has to specify the location of the Cytoscape executable. Afterwards, AGeNNT starts Cytoscape without further assistance.

## Results

### Function of AGeNNT

AGeNNT requires an SSN computed by the EFI-EST service (http://efi.igb.illinois.edu/efi-est/) as input. The computation of an SSN is detailed in [9] and demonstrated in Additional file 1. EFI-EST provides a set of output files containing networks of different granularity. If the network has less than 10 M edges, a full network is provided. Additionally, representative node (rep-node) networks are offered. In a rep-node *k* network, sequences that share at least *k*% identical resides are collected in the same sequence node. Thus, the number of nodes and edges as well as phylogenetic bias can be reduced by analyzing a rep-node 95 or a rep-node 80 network.

Due to their comprehensive annotation, we recommend the usage of SSNs deduced from InterPro families and the BLAST *E*-value cut-off 1E-5 in order to create most comprehensive networks. The results shown below are based on InterPro version 58 or 60. The outcome of the subsequently applied network clustering algorithm depends on the threshold *Th* applied to the edge weight distribution of the SSN. AGeNNT utilizes a previously proposed heuristic [7] and an in-house method to compute two alternatives, named *A-Th* and *S-Th*. After the user has selected a threshold, AGeNNT eliminates from the initial SSN all edges not reaching *Th* and initiates the computation of a GNN. To start this EFI service, the user has to specify the size of the genome neighborhood (*nb* between ±3 and ±10 genes), the co-occurrence lower limit (1 to 100%) and an email address. The co-occurrence lower limit is the fraction of sequences from the given sequence cluster that possess a gene-product encoding the functionality of a Pfam-node *p*_*l*_ within their *nb* neighborhood.

**Fig. 1:**
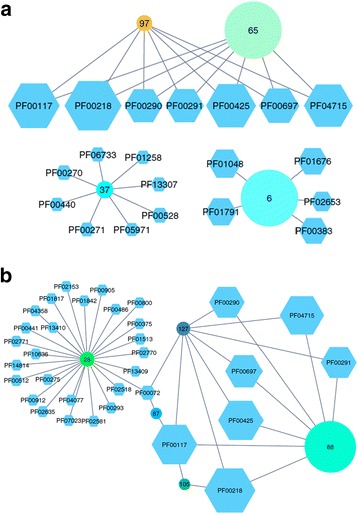
Excerpts of two rGNNs of IPR000312 (TrpD and homologs). **a** Using Cytoscape, all nodes with less than 150 sequences were deleted from the rGNN and the position of nodes was interactively altered for ease of interpretation. “Hubs” (*circles*) represent sequence clusters from the corresponding SSN and “spokes” (*hexagons*, labeled with Pfam-IDs) protein functions found within the respective GN. The diameter of the elements corresponds to the number of sequences. Clusters 6 and 37 possess distinct GNs that differ from the GN of clusters 65 and 97. Due to the parameters selected for SSN generation, these isofunctional sequences constitute two sequence clusters. Cluster 65 comprises sequences from different bacterial phyla; sequences of cluster 97 are mainly from Bacteroidetes. **b** This rGNN was created in the same manner as the one shown in (**a**). However, subspecies filtering was not applied. This is why this rGNN contains cluster 28, which is a highly specific GN of few proteobacterial species. Cluster 88 corresponds to cluster 65 shown in (**a**)

After the completion of the EFI service, AGeNNT downloads the GNN to the user’s computer and offers the visualization of the EFI GNN, a colored SSN, and the refined GNN (rGNN) by means of Cytoscape [10]. A GNN interlinks cluster-nodes (representing a sequence cluster of the corresponding SSN) and Pfam-nodes (representing the corresponding GNs). Each GN consists of those Pfam-nodes *p*_*l*_ that reach the specified co-occurrence lower limit. Similar to the sequence-centered version of the EFI GNNs, the “hubs” of the network are the cluster-nodes that are linked to “spokes” that are the Pfam-nodes. Due to this representation, it is easy to determine and to compare the GNs of different sequence clusters; compare Fig. 1a. In comparison with EFI-GNNs, rGNNs benefit from the following features that decisively support the annotation of enzymes:

- AGeNNT assists the user in specifying the threshold *th* required to filter the initial SSN prior to GNN generation; see Formula (3).
- To reduce graph complexity, AGeNNT optionally eliminates gene neighbors without enzymatic function based on an editable “whitelist” containing Pfam-IDs, which represent enzymatic functions.
- Each Pfam-node is additionally annotated with GO terms that specify the molecular function and the metabolic process in which the enzyme is involved.
- On demand, AGeNNT reduces phylogenetic bias. Then, each species can only contribute one sequence to each cluster node and the sequences from related subspecies are eliminated. To further characterize the phylogenetic distribution of the enzymes, AGeNNT determines the normalized frequencies of all phyla contributing to each cluster-node.
- The size of the nodes corresponds to the number of clustered sequences (*SeqCount*). Thus, dominant as well as more special GNs can be discriminated easily.
- For each edge, interconnecting a cluster node and a Pfam node, the *Coverage* is given, which is the relative number of genomes supporting this link of a cluster-node and an enzyme function (Pfam-node) encoded in the considered neighborhood.
- In order to assist the user in comparing GNs, AGeNNT computes statistics to assess the “uniqueness” of edges, Pfams, and GNs; see Formulae (4) to (7).

The usage of AGeNNT is detailed in Additional file 1. In the following, we motivate the computation of a novel threshold, show typical results and a novel application of rGNNs, and illustrate the predictive power of rGNNs by analyzing some retrospective test cases. The processed rGNNs of all test cases can be downloaded from our GitHub repository; see release tab.

### *S-Th*, a more adequate threshold for the analysis of dense SSNs

The aim of SSN generation is to distribute sequence-nodes among cluster-nodes that represent each a certain protein function, *i. e.* an isofunctional protein family. In order to make possible the clustering by means of graph analysis techniques, the edges interconnecting the sequence-nodes of an EFI SSN are labeled with scores indicating the pairwise similarity determined by BLAST [11]. It turned out that the performance of network clustering algorithms depends on thresholding the edges prior to clustering [7] and for each network a proper threshold *Th* has to be found. If chosen correctly, *Th* separates inter-family edges from intra-family edges [7]. For the assessment of their threshold (*A-Th* value), the authors had analyzed four sequence sets consisting at most of 1308 sequences, each representing the full diversity of a protein family [7]. Due to the low pairwise similarity of many sequences, the corresponding SSNs were sparse networks, which is no longer the case for current datasets. Nowadays, most datasets representing protein families contain large numbers of sequences, which are often highly similar to each other. Thus, the resulting SSNs are dense networks, as the number of edges is usually at least ten fold higher than the number of nodes. To illustrate this circumstance, Table 1 lists characteristic values of the five InterPro families analyzed below. As a consequence, the *A-Th* value is often chosen too high for a representative analysis. Therefore, we suggest an alternative, smoothed threshold named *S-Th* (Formula (3)), which eliminates more edges; see Table 1.

**Table 1:** Characteristics of InterPro families and resulting SSNs

InterPro family	# seq	Rep-node 100				Rep-node 80			
		# nodes	# edges	*A-Th*	*S-Th*	# nodes	# edges	*A-Th*	*S-Th*
IPR000312	21,626	17,712	71,376,439	190	107	5446	6,789,466	195	97
IPR004651	10,868	8521	36,297,962	140	101	1830	1,672,923	104	88
IPR023016	9463	7428	27,581,256	144	78	1920	1,842,066	114	67
IPR015890	29,878	14,614	96,362,962	259	96	8388	31,307,224	259	86
IPR007115	10,421	7848	12,511,920	70	40	2901	1,609,102	57	34

The first column gives the name of the InterPro family and the second one the number of sequences belonging to this dataset. The four columns entitled Rep-node 100 and Rep-node 80, respectively, list the number of nodes and edges of the corresponding SSN and the thresholds *A-Th* and *S-Th*. For the generation of the dataset, the BLAST *E*-value cut-off 1E-5 was used

### An rGNN of the glycosyl transferase family

IPR000312 is named glycosyl transferase family 3 and subsumes all enzymes that transfer a phosphorylated ribose substrate. The family includes anthranilate phosphoribosyltransferases (TrpD, EC 2.4.2.18) and thymidine phosphorylases (DeoA, EC 2.4.2.4). To create Fig. 1a, we chose the proposed *S-Th* value of 97, eliminated subspecies and created an rGNN by collecting ±10 neighbors at a co-occurrence value of 20%. For visualization, we used Cytoscape and eliminated all nodes representing < 150 sequences. For all representations of rGNNs shown below, Cytoscape’s organic layout was applied initially and the position of nodes was rearranged interactively for ease of interpretation.

The comparison of the neighborhoods makes clear that the homologs constituting IPR000312 possess at least three different enzymatic functions: The GN of clusters 97 (362 sequences) and 65 (3858 sequences) comprises PF00117, PF00218, PF00290, PF00291, PF00425, PF00697, and PF04715, which represent gene products of the canonical tryptophan operon surrounding *trp*D. For the edges interconnecting cluster-node 97 and Pfam-nodes, all *Coverage* values are > 0.72, for cluster 65 the values are in the range between 0.4 and 0.6. These numbers indicate a lower GN conservation for the physiologically diverse TrpD sequences which are mainly from Proteobacteria and Firmicutes clustered in node 65, in contrast to the GNs of the physiologically more related TrpD sequences of cluster 97, which are mainly from Bacteroidetes. The GN of cluster 6 (3834 sequences) represents a typical GN of nucleoside phosphorylases (DeoA). The 452 sequences of cluster 37 are annotated as glycosyl transferases. However, the GN contains two domains of a DNA unwinding helicase (PF00271, PF06733) suggesting DNA interaction for these 452 sequences. Indeed, one representative, YbiB, has recently been characterized as a DNA-binding protein [12]. Altogether, this retrospective test case demonstrates the predictive power of GNNs, because the GNs are indicative of putative functions for all three clusters of homologs.

The number of Pfam-nodes belonging to these GNs is relatively small, because the elimination of sequences from subspecies removed any species-specific conservation of gene arrangements. Figure 1b is part of an rGNN created with the same parameters albeit lacking subspecies elimination. This rGNN contains the cluster-node 28 representing 661 sequences mostly from Proteobacteria. The corresponding SSN makes clear that these sequences are combined in not more than 60 sequence nodes and the three most populated ones represent 228, 173, and 73 sequences originating predominantly from subspecies. Due to their close phylogenetic relationship, their genome neighborhood is highly conserved. This is why the GN of cluster 28 contains 25 additional Pfam nodes that occur as neighbors in the genomes of few proteobacteria. The uniqueness value of cluster-node 28 is 6667, which also indicates a highly specific combination of enzyme functions.

This example illustrates that the elimination of phylogenetic bias helps to avoid GNs whose content is overly specific due to a close phylogenetic relationship. Such GNs can be misleading, if the ±10 neighborhoods contain enzymes from different operons which commonly show no strong functional coupling. On the other hand, the GN of cluster 88 indicates the robustness of rGNN topology: As expected, this dominating GN represents the tryptophan operon. It consists of PF00117 (TrpG), PF00218 (TrpC), PF00290 (TrpA), PF00291 (TrpB), PF00425 (TrpE, catalytic domain), PF00697 (TrpF), and PF04715 (TrpE, N-terminal region). This set is identical to the GNs of clusters 65 and 97 of Fig. 1a. The numbering of the clusters is different, because the subspecies filter alters the composition of the sequence sets. Figure 1a is lacking a GN corresponding to cluster 28 of Fig. 1b, because after subspecies elimination, the respective sequence nodes contained less than 150 sequences each and were eliminated.

In order to further demonstrate the robustness of rGNNs, especially with respect to the parameters chosen for their generation, we created 27 rGNNs based on the rep-node 100, rep-node 80, and rep-node 60 files of IPR000312 and varied in a systematic manner the neighborhood (±3, ±6, and ±10) and the co-occurrence (10, 20, and 30%) values. The corresponding Figures S1–S3 can be found in Additional file 2. To allow for a straightforward comparison of the networks, the same color code was used for all plots and all graphs were oriented the same way. A comparison of the networks makes clear that – as expected – the complexity is highest for the rep-node 100 network analyzed with a ±10 neighborhood and a 10% co-occurrence. For each rep-node *k* dataset, the complexity of GNs decreases for a smaller neighborhood and a higher co-occurrence value. Moreover, complexity goes down with the decrease of the *k* value. However, as can be seen, characteristic Pfam-nodes like (PF00117, PF00218), (PF00383, PF01791), and (PF00271, PF06733), which are important for functional assignment of cluster-nodes representing TrpD, DeoA, or YbiB–like enzymes are present in all networks.

In summary, this systematic analysis of one InterPro family testifies to the robustness of the SSN/rGNN approach, because the assignment of three specific enzyme functions is possible for a broad range of parameter combinations.

### The GNN of a monofunctional enzyme has a simple topology

**Fig. 2:**
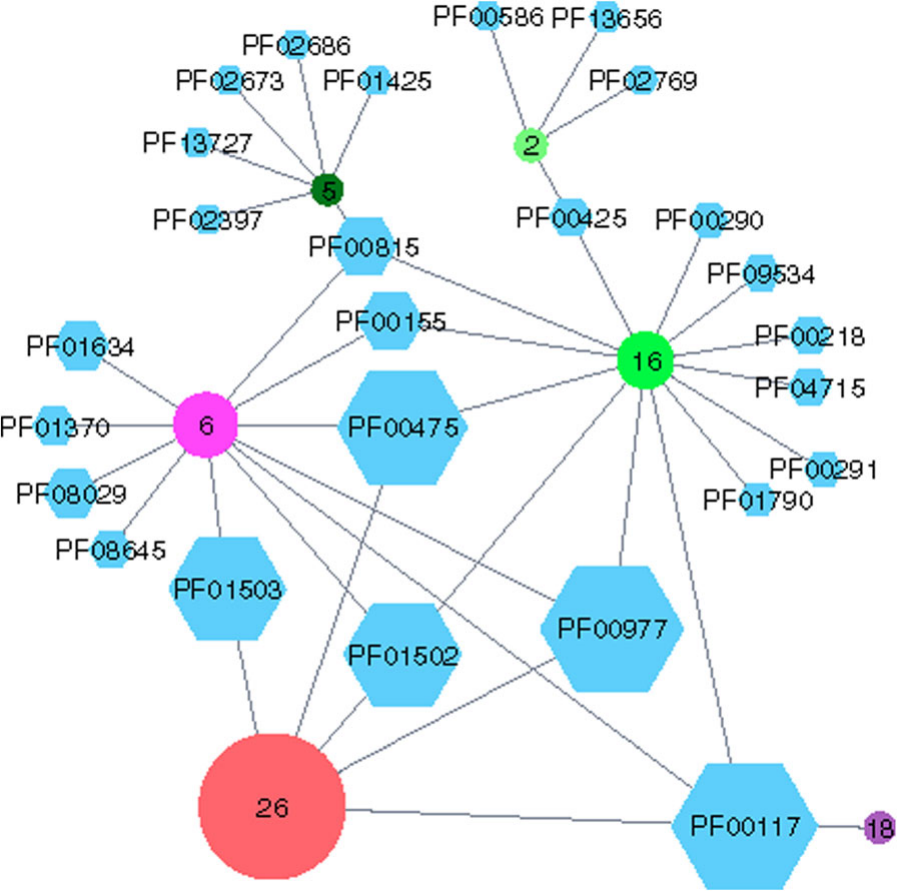
rGNN of IPR004651 (HisF). The biosynthesis of histidine requires reactions catalyzed by eight enzymes. HisF catalyzes the cyclization reaction needed for the sixth step. This rGNN was created by applying the *S-Th* threshold of 97, subspecies were eliminated, and ±10 neighbors at a co-occurrence value of 20% were collected. All nodes with a *SeqCount* value ≤ 25 were deleted

*his*F is encoded in the histidine operon of Bacteria and its gene product catalyzes a cyclization reaction during the sixth step of histidine biosynthesis. For bacterial HisF, no further function has been described. In order to illustrate how adjacent operons affect the composition of GNs, we computed an SSN for the InterPro family IPR004651 with a BLAST *E*-value of 1E-5 by means of the EFI-EST service. We applied the threshold *S-Th*, which was 97, and eliminated subspecies. An rGNN was created by collecting ±10 neighbors at a co-occurrence value of 20%. The number of sequences belonging to different nodes varied from 1 to 3574. To eliminate low populated nodes, all nodes with a *SeqCount* value ≤ 25 were eliminated. Figure 2 shows the resulting rGNN; interestingly, it contains not more than six cluster-nodes.

Cluster node 26 (uniqueness 43) represents 3574 sequences from different phyla. The corresponding GN consists of PF00117 (glutamine amidotransferase class I), PF00475 (imidazoleglycerol-phosphate dehydratase), PF00977 (histidine biosynthesis protein), PF01502 (phosphoribosyl-AMP cyclohydrolase) and PF01503 (phosphoribosyl-ATP pyrophosphohydrolase). These enzymes constitute the core of the canonical histidine operon and the two smaller clusters 6 (uniqueness 1345, 1040 mainly proteobacterial sequences) and 16 (uniqueness 1054, 813 bacterial sequences with a large fraction of Actinobacteria) possess in their specific GNs these Pfam-nodes as well. Clusters 6 and 16 share in their GNs PF00155 (aminotransferase class I and II) and PF00815 (histidinol dehydrogenase), which are also involved in histidine biosynthesis, but are seemingly less conserved in bacterial operons. Two Pfam-nodes specific for cluster 6, namely PF08029 and PF01634, are domains of the ATP phosphoribosyltransferase, which is also related to histidine biosynthesis. Most likely, the remaining two enzymes PF08645 (polynucleotide kinase 3 phosphatase) and PF01370 (NAD dependent epimerase/dehydratase) are not involved in histidine biosynthesis but are co-located.

Interestingly, five of the seven enzyme functions specific for cluster 16 are related to tryptophan biosynthesis. These are PF00218 (indole-3-glycerol phosphate synthase, TrpC), PF00290 (tryptophan synthase alpha chain, TrpA), PF0291 (pyridoxal-phosphate dependent enzyme, TrpB), PF00425 (TrpE, catalytic domain), and PF04715 (Trp E, N-terminal region). Inspecting the HisF neighborhood by using the *Genome Browser* of the BioCyc server [13] shows that the histidine and tryptophan operons of Mycobacteria and other Actinobacteria are directly adjacent in their genomes. This is why the GN of cluster 6 contains functions from two operons. Cluster 2 (uniqueness 634) represents 76 sequences dominantly from Euryarchaeota, cluster 5 (uniqueness 1093) contains 45 sequences from Spirochaetes, and cluster 18 (uniqueness 8) 44 sequences mainly from Proteobacteria. Without deeper analysis, these marginally populated GNs are difficult to interpret. In contrast, the composition of the three dominating GNs shows that the rGNN of a mono-functional enzyme has a relatively simple topology.

### Correlating enzymatic function of HisA and PriA and the localization of their genes

For two actinobacterial species, namely *Streptomyces coelicolor* and *Mycobacterium tuberculosis*, the existence of an enzyme named PriA has been reported [14]. With respect to sequence and structure, PriA is highly similar to HisA, which catalyzes the isomerization of an aminoaldose in histidine biosynthesis. Interestingly, Actinobacteria lack a *trp*F gene and it turned out that PriA is a bi-functional homolog of HisA, which adopts the roles of HisA in histidine and of TrpF in tryptophan biosynthesis. The evolution of PriA is unclear; it has been suggested that its bi-functionality is an evolutionary response to the loss of the *trp*F gene and that a narrowing down of the PriA specificity in certain Actinobacteria occurred after the horizontal gene transfer of a whole *trp* operon [15]. An SSN of the HisA/PriA superfamily (IPR023016) made clear that *his*A genes are present in all major phylogenetic groups and that the occurrence of annotated *pri*A genes is indeed restricted to the Actinobacteria. Moreover, by characterizing ancestral HisA enzymes, it was made plausible that HisA has been a bi-functional enzyme for at least 2 billion years, most likely without any evolutionary pressure [16]. The latter results are strong evidence for the assumption that PriA is a typical HisA successor due to the bi-functionality of the ancestral HisA enzymes.

**Fig. 3:**
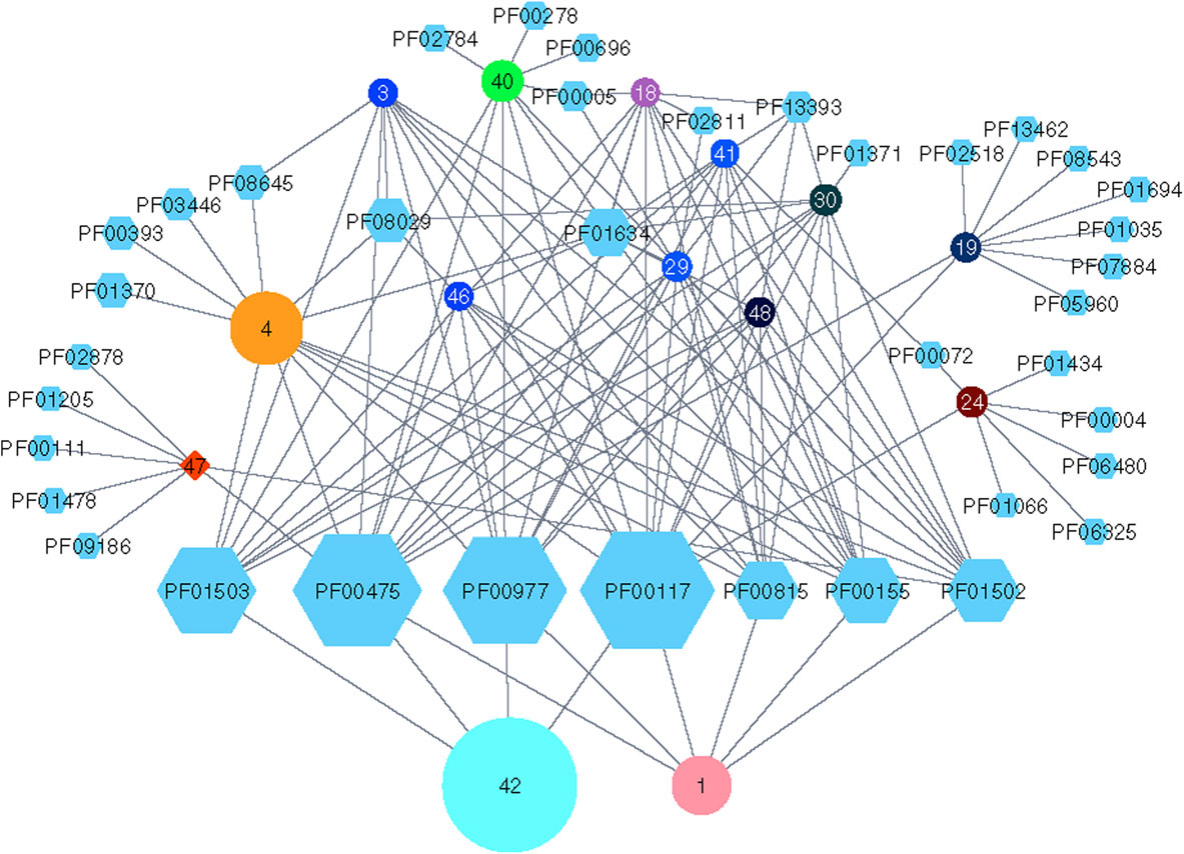
rGNN of IPR023016 (HisA/PriA). PriA is a bifunctional enzyme that adopts the roles of both HisA in histidine and of TrpF in tryptophan biosynthesis. Cluster-node 1 contains the actinobacterial HisA homologs (PriA) and cluster-nodes 4 and 42 homologs from different Bacteria. The GNs of all three cluster-nodes overlap to a great extent and consist of enzymes involved in histidine biosynthesis. The Pfams belonging to the overlap of GNs are arranged in a line

If one can show that the GN of bi-functional PriA enzymes is highly similar to the GN of typical HisA enzymes, one can further confirm that PriA is more a typical than an exceptional HisA homolog. Figure 3 shows the rGNN determined for IPR023016. Using the EFI-SSN service and an *E*-value of 1E-5 as BLAST cut-off, an SSN was created. The *S-Th* value of 75 and elimination of subspecies was chosen prior to the generation of the rGNN. For highest sensitivity, a ±10 neighborhood was analyzed; however, in order to eliminate enzymes belonging to the adjacent tryptophan operon (compare Fig. 2), a co-occurrence of 25% was used to compute the rGNN. Using Cytoscape, all cluster-nodes representing ≤ 20 sequences were deleted. Besides few outliers, all HisA homologs from Actinobacteria belong to sequence-cluster 1 that represents 579 sequences. Cluster-nodes 4 (821 sequences) and 42 (1946 sequences) comprise sequences from different bacterial phyla. The GNs of these three clusters overlap to a great extent and consist of enzymes involved in histidine biosynthesis. Most importantly, the GN of the actinobacterial HisA homologs does not contain enzyme functions not found in the other GNs of bacterial HisA enzymes. This finding makes clear that with respect to functional coupling, the actinobacterial PriA enzymes do not differ from other HisA homologs.

### The function of chorismate-utilizing enzymes can be deduced from their specific GNs

The sequences of the C-terminal domain of chorismate-utilizing enzymes constitute IPR015890. Among these enzymes are anthranilate synthases (AS), aminodeoxychorismate synthases (ADCS), isochorismate synthases (ICS), and salicylate synthases (SS). AS and ADCS catalyze mechanistically related reactions using ammonia as a nucleophile in tryptophan and folate biosynthesis, respectively. Although ICS and SS are highly similar to AS and ADCS with respect to sequence and structure, they utilize water instead of ammonia as a nucleophile and are part of secondary metabolic biosynthetic pathways leading to iron-chelating siderophores (e. g. enterobactin, yersiniabactin, and mycobactin) and electron-transport compounds (menaquinone).

It has recently been shown that only two amino acid substitutions in AS are sufficient to generate a bi-functional enzyme that forms isochorismate as efficiently as does a native ICS while retaining AS activity [17]. Thus, the comparison of sequences in this enzyme family may be misleading and the determination of function from sequence homology may be erroneous. However, as these enzymes are part of different metabolic pathways, their GNs should be indicative of their predominant function.

**Fig. 4:**
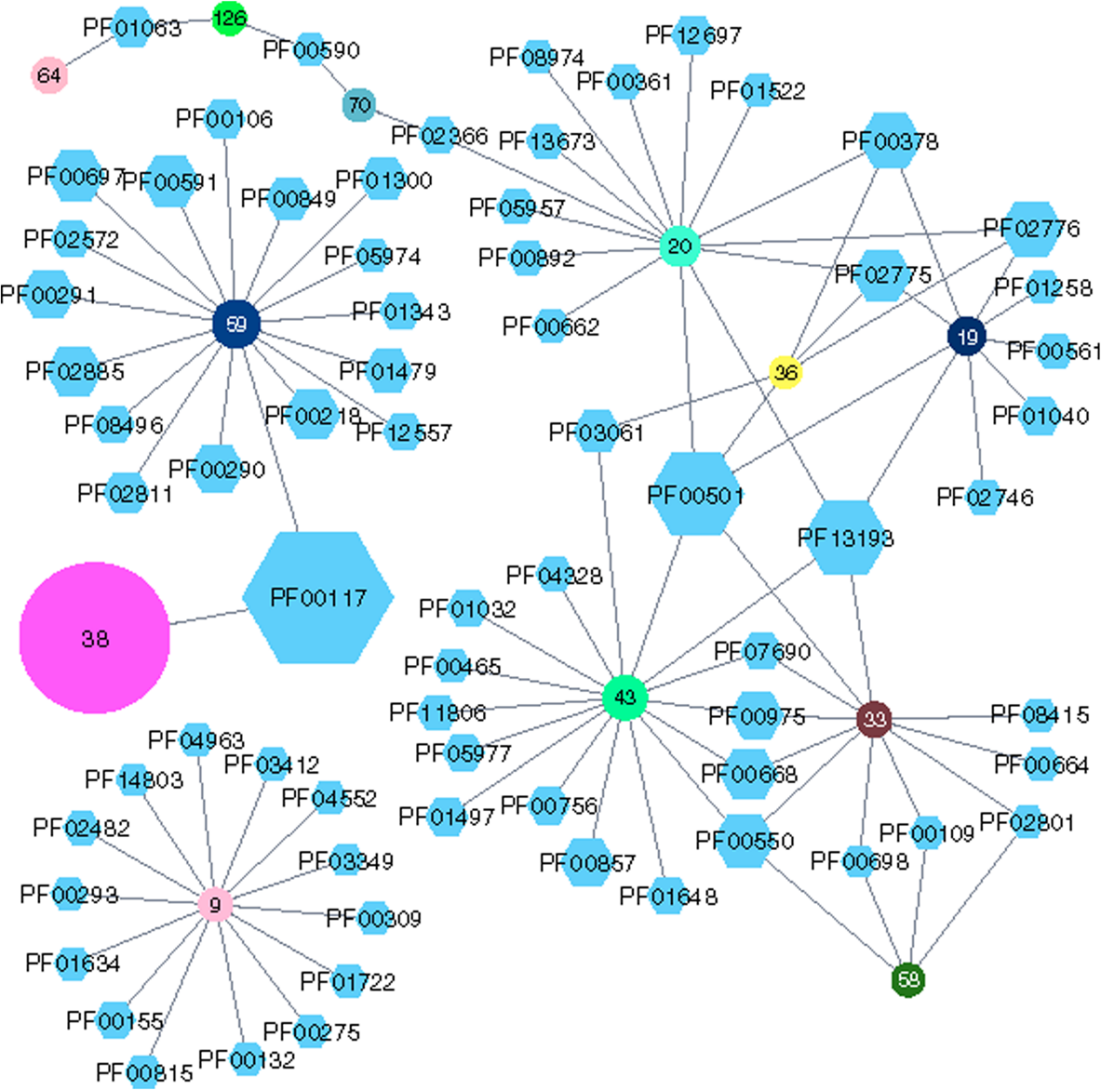
rGNN of IPR015890 (chorismate-utilizing enzymes, C-terminal domain). The family of chorismate-utilizing enzymes comprises several homologous but functionally diverse enzymes from different primary and secondary metabolic pathways. The enzyme AS from tryptophan biosynthesis is represented by cluster-nodes 38 and 59, whose GN represents the typical tryptophan operon. The GN of ADCS from folate biosynthesis (cluster-node 64) is less conserved and consists of one Pfam-node, representing the enzyme catalyzing the subsequent step of folate biosynthesis. The homologous ICS and SS enzymes are part of different biosynthesis pathways that lead to iron-chelating siderophores as well as electron transport compounds. The rGNN greatly assists in separating these enzymes into different isofunctional groups: The GNs make clear that cluster-nodes 20, 36, and 19 represent MenF-type ICS, cluster-node 43 represents EntC-type ICS, and cluster-node 33 represents SS enzymes

The rGNN of this enzyme family (Fig. 4) was computed using the EFI-SSN service and an *E*-value of 1E-5 as BLAST cut-off. The *S-Th* value of 93 and elimination of subspecies was selected prior to the generation of the rGNN with a ±10 neighborhood and a co-occurrence of 20%. Using Cytoscape, all cluster-nodes representing ≤ 100 sequences were eliminated and the organic layout was utilized for visualization.

Dominating is cluster-node 38, which represents 8191 AS and ADCS sequences from several major bacterial and archaeal phyla. Its GN consists of only one Pfam-node, PF00117 (glutamine amidotransferase class I) that subsumes the glutaminases TrpG and PabA, which are part of the heterodimeric AS and ADCS complexes and deliver the ammonia required for the AS and ADCS reactions. Most likely, the occurrence of AS and ADCS enzymes in many, phylogenetically less related species and the variability of the genome neighborhood is the reason that this GN consists of not more than the glutaminase subunits. PF00117 also belongs to the GN of cluster-node 59 which additionally contains 16 other Pfam-nodes. The respective sequences are almost exclusively of proteobacterial origin and this close relationship is the reason that this GN contains other enzymes of the *trp* operon, namely PF00218 (TrpC), PF00697 (TrpF), and PF00290 (TrpA). The GNs of other ADCS also seems to be highly diverse, the main ADCS cluster, cluster-node 64, only contains PF01063 (amidotransferase class IV) which represents PabC, the enzyme that catalyzes the subsequent step in folate biosynthesis.

Cluster-node 9 is special due to a unique GN consisting of 14 Pfams. The sequences comprising this cluster are almost exclusively from *Acinetobacter* and *Psychrobacter* species and are annotated as either AS or ADCS. However, the GN does not contain a single enzyme typical for tryptophan or folate operons but instead contains several DNA- or ATP-binding proteins, hydrolases, and a histidinol dehydrogenase. Presumably, the sequences of cluster-node 9 represent homologous copies of AS or ADCS that are part of a different functional context in *Acinetobacter* and *Psychrobacter* and they may be interesting candidates for elucidating enzymatic function.

As mentioned above, IPR015890 also contains ICS and SS. These two enzymes are highly similar in sequence and structure and their respective reactions differ only in the different processing of their common product. Thus, false annotations are very common for ICS and SS. Moreover, several isozymes of ICS are known that catalyze the same reaction in different metabolic contexts. For example, the ICS EntC is part of the biosynthesis of the siderophore enterobactin, whereas the ICS MenF is part of the biosynthesis of menaquinone. The rGNN can help to reliably assign the ICS sequence of this enzyme family to the different isotypes. For example, the cluster-nodes 20, 36, and 19 represent ICS sequences from Proteobacteria, Firmicutes, and Bacteroidetes, respectively, and share five Pfams in their GNs (PF00378, PF02775, PF02776, PF03061, and PF00501). However, the GN of cluster-node 19 exclusively contains PF01040, which represents the UbiA prenyltransferase family. One homolog of this family, MenA, is part of the biosynthesis of menaquinone [18]. Along the same lines, the GN of cluster-node 20 exclusively contains PF00662, which represents a family of NADH-ubiquinone oxidoreductases, some of which also accept menaquinone as an electron acceptor [19].

Cluster-node 43 represents the other isotype of ICS, EntC. Its GN contains PF00975 (thioesterase domain) and PF00668 (condensation domain), which are part of the non-ribosomal peptide-synthetases that catalyze the formation of enterobactin-type siderophores [20]. Moreover, this GN contains PF00857 (EntB), the enzyme that catalyzes the step following the EntC reaction in the biosynthesis of enterobactin. Cluster-nodes 43 and 33 have in common four Pfams in their respective GNs (PF00975, PF00550, PF07690, and PF00668). However, the GN of cluster-node 33 does not contain PF00857 (EntB), which supports the annotation of these sequences as SS. In contrast to ICS, these enzymes directly convert chorismate to salicylate and not via an additional step (catalyzed by EntB) to 2,3-dihydro-2,3-dihydroxybenzoate.

In summary, the analysis of the InterPro family of chorismate-utilizing enzymes illustrates how GNs in combination with the functionality of AGeNNT can help to reliably dissect large families of homologous enzymes with diverse functions and different metabolic contexts.

### rGNN analysis immediately reveals at least three different functions of PTPS enzymes

The InterPro entry IPR007115 is named “6-pyruvoyl tetrahydropterin synthase/QueD family protein” and contains 10,421 sequences that possess 18 different domain architectures [1]. 6-pyruvoyl tetrahydropterin synthase (PTPS) catalyzes the conversion of dihydroneopterin triphosphate to 6-pyruvoyl tetrahydropterin, which is the second of three enzymatic steps in the synthesis of tetrahydrobiopterin from GTP [21]. The enzyme QueD, which contributes to the biosynthesis of queuosine, a hypermodified base in the wobble position of some tRNAs in bacteria and eukaryotes [22], is also part of this functionally diverse superfamily [23]. In total, at least six PTPS homologs with different enzymatic functions named PTPS-I – PTPS-VI have been described to date, which are also often misannotated and hard to discern via sequence similarity alone [24, 25]. Moreover, several bacterial species possess more than one PTPS homolog and a standard GNN approach did not allow the accurate and precise identification of the different enzymatic functions [25]. We expected a better performance of an rGNN analysis due to the following reasons:

1. AGeNNT’s built-in filters reduce noise and eliminate Pfam-nodes less relevant for a functional assignment.
2. AGeNNT’s representation of GNs allows the detection of clearly distinct GNs but also function-related overlaps between clade-specific GNs.

**Fig. 5:**
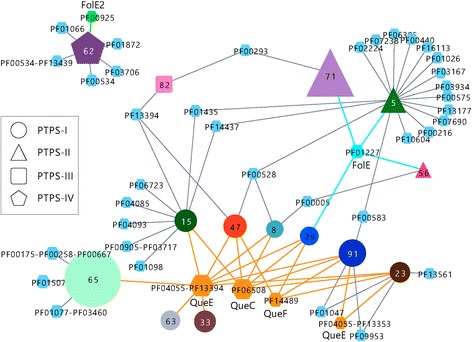
rGNN of IPR007115 (6-pyruvoyl tetrahydropterin synthase/QueD family protein). This rGNN (named rGNN_7115_150) contains all nodes that represent more than 150 sequences. Dominating are cluster-nodes 65 (PTPS-I enzymes), 71 (PTPS-II enzymes), and 62 (PTPS-IV enzymes). Dominating Pfam-nodes are related to queosine biosynthesis (QueC, QueE, and QueF). The GNs of PTPS-II enzymes (cluster 71) and of PTPS-III enzymes consist of not more than two Pfam-nodes. The GNs of PTPS-IV enzymes contain FolE2; FolE belongs to the GN of cluster-nodes 5, 56, and 71

To begin with, we computed an rGNN for IPR007115 based on the rep-node 80 file and used our standard parameters, *i. e*., the BLAST *E*-value cut-off 1E-5, a ±10 neighborhood, and a co-occurrence of 20%. Additionally, we applied the *S-Th* value of 34 and eliminated subspecies. By utilizing Cytoscape’s *Select* command, we generated the network named rGNN_7115_150 that contains all cluster- and Pfam-nodes representing at least 150 sequences; see Fig. 5.

Dominant elements of rGNN_7115_150 are three cluster-nodes (62, 71, and 65) that possess distinct GNs. The GN of cluster 71 (uniqueness 182, 1183 sequences) contains not more than two Pfam-nodes, namely PF01227 (GTP cyclohydrolase I; including the folate biosynthetic enzyme FolE) and PF00293 (nudix family proteins that hydrolyze a wide range of organic pyrophosphates). Interestingly, cluster-node 62 (uniqueness 1629, 561 sequences) also contains a GTP cyclohydrolase I, namely PF00925 (GTP cyclohydrolase I; includes the folate biosynthetic enzyme FolE2), but also PF01872 (RibD C-terminal domain) which is involved in riboflavin biosynthesis [1]. Cluster 65 (uniqueness 1046) represents 1463 sequences mainly from Proteobacteria. The most prominent Pfam-node of the corresponding GN is PF04055-PF13394 (radical SAM superfamily-4Fe-4S single cluster domain that includes the queosine biosynthesis enzyme QueE).

This Pfam-node occurs in nine GNs and the corresponding cluster-nodes (65, 15, 47, 8, 29, 91, 23, 63, and 33) represent a total of 4065 sequences. The GNs of these clusters also contain PF06508 (QueC), PF14489 (QueF) and PF4055-PF13353 (QueE), all of which contribute to queosine biosynthesis. Thus, the abundance of sequences and the density of the network make clear that QueD (PTPS-I) functionality is most prevalent in this InterPro family. As a first result, we postulate at least three different enzyme functions for PTPS homologs; the analysis of a conventional GNN did not allow such a classification [25].

In order to illustrate a more profound analysis of a complex case, we generated a second, more detailed network named rGNN_7115_30 that contains all cluster- and Pfam-nodes representing at least 30 sequences; see Additional file 2: Figure S4. Additionally, we analyzed sequence motifs, which are indicative of the functionality of PTPS homologs [24, 25] and can easily be deduced for cluster-nodes; see the protocol given in the legend of Additional file 2: Figure S4. As expected, the concerted analysis of known motifs and GNs allows a more precise specification of PTPS functions: For example, the clusters predicted as being involved in queosine biosynthesis showed the motif CxxxHGH that is typical for PTPS-I functionality [25]. All sequences of cluster-node 71 contain the PTPS-II typical sequence motif CxxxxxHGH [25]. In contrast, the sequences of cluster-nodes 5 and 56 contain the motifs CxxxxHGH and CxxxHGY, thus we termed them PTPS-II-like. The sequences of cluster-node 82 share the motif ExxHGH indicative of PTPS-III functionality and those of cluster-node 62 preferentially contain the PTPS-IV-typical sequence signature FGPAQ [25].

Importantly, our rGNN approach identifies FolE2 as element of the PTPS-IV neighborhood, which has not been recognized previously, possibly due to the low number of analyzed sequences. Moreover, our analysis of more than 10,000 sequences suggests a highly variable GN of PTPS-II sequences (cluster-node 71); the coverage of the edge interconnecting cluster-node 71 and PF01227 is not higher than 0.13. A more detailed analysis of rGNN_7115_30 can be found in the legend of Additional file 2: Figure S4.

Taken together, by analyzing this InterPro family of PTPS homologs, we have demonstrated that the visualization of GNs as interwoven networks helps to corroborate similarities and differences of GNs. By eliminating smaller GNs, the user can create a “bird eye’s view” on enzyme interactions and identify those ones that are conserved in many, phylogenetically distinct genomes. These results nicely supplement findings deduced from the specific analysis of individual genomes and are additionally robust against the effect of “genomic hitchhiking”. Most likely, some microbial neighborhoods are merely due to the expression level and not to the functional theme of a given neighbourhood [26], which makes some individual cases enigmatic.

## Discussion

### Potentials and limitations of SSNs, GNNs, and rGNNs

The exponential growth of sequence data demands the development of robust and simple to use techniques to support the experimental biologist in analyzing and restructuring functionally diverse enzyme superfamilies. SSNs are an easy to use alternative to multiple sequence alignments (MSAs) and phylogenetic trees. For large and diverse sequence sets, it is difficult to construct a reliable MSA. This in turn impedes the identification of sequence motifs that are specific for the different functions of homologs and also the computation of a phylogenetic tree. On the other hand, the two-dimensional distances used in SSNs represent much of the information underlying phylogenetic trees [27]. Moreover, when analyzing and annotating large superfamilies, the main goal is not to create the optimal representation of sequence similarity, but to allow the user the visualization of many protein attributes that are orthogonal to sequence similarity and represent derived information. As has been demonstrated, by mapping these features onto SSNs by means of interactive software like Cytoscape, the informed user can rapidly develop hypotheses about the function of family members [16, 27–33].

The synergistic use of SSNs and GNNs further assists the user in assigning enzyme function, because the genome neighbors are expected to be functionally related to the enzymes under study. Along this line, experiments confirmed that the majority of the 2333 enzymes of the proline racemase superfamily catalyze only three known reactions. Thus, by using GNNs without additional information, the function of > 85% of the family members could be predicted [34].

We made plausible that the simultaneous analysis of many phylogenetically unrelated genomes reduces the risk of creating overly specific GNs containing functionally unrelated enzymes. To the best of our knowledge, the EFI services are the only algorithms that deduce and combine GNs for a large number of homologous protein sequences. In contrast, alternatives like the *Multi-Genome Browser* of *BioCyc* [13] or PSAT [35] are focusing on the analysis of few genes or are restricted to co-expressed genes [36] or mammalian genomes, like G-Nest [37]. Thus, the strength of EFI GNNs is the possibility to deduce GNs from many genomes and to combine them. Moreover, these GNNs are an ideal representation of the commonalities and differences of GNs found for homologous proteins.

AGeNNT provides additional guidance in functional annotation. However, the user has to anticipate several kinds of bias that may affect the composition of the resulting rGNNs:

1. In contrast to initial implementations of GNs [4], the EFI GNNs comprise all protein functions encoded by neighboring genes irrespective of their orientation and their regulation. Thus, a GN may contain functionally unrelated gene-products belonging to different operons. This risk is higher, if a large neighborhood value was chosen for GNN compilation and if the operon under study consists of only few genes.
2. If some species (and subspecies) are overrepresented in a cluster-node, their species-specific GN can dominate the GN determined for the whole cluster-node.
3. The composition of the individual GNs depends on the clusters constituting the SSN.

A combination of enzyme functions from several operons to the same GN (problem 1) is more likely for cluster-nodes representing closely related species but unlikely for less related ones, because the genome location of operons is not conserved on the grand scale. By inspecting the *PhylumStat* value computed by AGeNNT for each cluster-node, the user can deduce the phylogenetic relationship of the corresponding species.

Eliminating phylogenetic bias (problem 2) is difficult; however, the subspecies filter offered by AGeNNT and the usage of rep-node networks enables the user to purge a fair amount of this bias. Additionally, high uniqueness values are indicative of highly specific GNs related to a certain evolutionary environment or taxon. If accompanied by a broad phylogenetic spread shown by *PhylumStat*, small uniqueness values signal GNs that are found in many, less related taxa.

In order to assess the robustness of the findings deduced by means of rGNNs (problem 3), we recommend to utilize several parameter combinations and to compare the outcome. For example, if the parameters chosen for SSN generation give rise to a large “super cluster” consisting of several clusters *c*_1_ .. *c*_*n*_, the GN deduced from the corresponding rGNN might be indicative of only one cluster *c*_*j*_ but not for all of them. Moreover, if a genome contains two copies of a gene, the GNN is a composite of two genome neighborhoods. To identify such cases, the user can control the *SeqCount* and *Coverage* values to be found in the *Edge Table* of Cytoscape’s *Table Panel*. These values give for all edges that interconnect a cluster-node and a Pfam-node the absolute and relative number of genomes supporting this link. If these values are relatively small, the corresponding enzyme function occurs only in a fraction of clustered GNs.

## Conclusions

For monofunctional enzymes found in many phyla, the GN is conserved to a great extent and after filtering, the rGNN is of low complexity because adjacent operons vary in their composition. rGNNs of high complexity are indicative of homologous enzymes possessing different functions. The representation of these GNs generated by AGeNNT assists the user in discriminating highly specific genome content and a more common functional coupling of enzymes deduced from a large number of phylogenetically less related species. The rich annotation of cluster-nodes supports the user in structuring large protein families and in deducing putative functions based on the differing metabolic contexts.

## Additional files

Additional file 1: Usage of AGeNNT. A tutorial guiding through the process of generating SSNs, rGNNs by means of AGeNNT and their visualisation by means of Cytoscape. (PDF 3996 kb)

Additional file 2: Figures S1–S4. Representations of 27 rGNNs resulting from a systematic variation of parameters used to generate them and of rGNN_7115_30. (PDF 280 kb)

## Abbreviations

EFI: Enzyme Function Initiative
GN: Genome neighborhood
GNN: Genome neighborhood network
NCBI: National Center for Biotechnology Information
Pfam: Protein family
rGNN: refined genome neighborhood network
SSN: Sequence similarity network
Th: Threshold
