# Supplementary material for: AGeNNT: annotation of enzyme families by means of refined neighborhood networks
Source: BMC Bioinformatics. 2017 May 25;18:274. doi: 10.1186/s12859-017-1689-6 (PMC5445326; doi:10.1186/s12859-017-1689-6)
Supplement: Supplementary file 1 — Usage of AGeNNT. A tutorial guiding through the process of generating SSNs, rGNNs by means of AGeNNT and their visualisation by means of Cytoscape. (PDF 3996 kb) [file 12859_2017_1689_MOESM1_ESM.pdf]

AGeNNT: annotation of enzyme families by means of refined neighborhood networks

Florian Kandlinger[1,2], Maximilian G. Plach[1] and Rainer Merkl[1*]
[1]Institute of Biophysics and Physical Biochemistry, University of Regensburg,
D-93040 Regensburg, Germany

[2]Faculty of Mathematics and Computer Science, University of Hagen,
D-58084 Hagen, Germany

Tutorial

Creating an rGNN for IPR000312 (glycosyl transferase family)

Prerequisites: Installation of AGeNNT and Cytoscape

We assume that the user has downloaded the file https://www.bioinf.uni-regensburg.de/downl/agennt.zip and installed AGeNNT. Fur future versions of AGeNNT, we will supply an updated tutorial as part of agennt.zip.

Cytoscape, which is required for visualization, can be downloaded from http://www.cytoscape.org/. For Windows operating systems during the first call of Cytoscape within AGeNNT, the user has to specify the location of the Cytoscape executable. Afterwards, AGeNNT starts Cytoscape without further assistance. For Linux/UNIX operating systems the path to Cytoscape can be specified in the *settings.ini* file, which is located in *$HOME/agennt*.

In the following, the generation of an rGNN is explained in detail for the InterPro entry IPR000312. Initial and final datasets are part of the repository accompanying this tutorial.

Using the EFI enzyme similarity tool to create an SSN

The first step is to use the EFI service Enzyme Similarity Tool (http://efi.igb.illinois.edu/efi-est/) in order to create an initial dataset for an SSN. For this tutorial, we use *Option B* and enter the name of the family IPR000312 in the corresponding field, see Fig. T1.

After having entered a valid email address, the generation of the SSN dataset can be started by clicking the *GO* button. Note that in this case, InterPro Version 60.0 and UniProt Version 2016_10 as well as default parameters were used. The default for the BLAST E-value cut-off is 1E-5. The EFI service sends two emails, one at the beginning of dataset generation and a second one when the dataset is available. The latter mail contains a link to a webpage that offers a series of histograms deduced from the input data and three input fields that control the next step of SSN generation. For this tutorial, we use as a lower limit for the alignment score X = 5 that corresponds to a cut-off of 1E-5 and use "IPR000312_E-5" as *Network Name*; see Fig. T2. After clicking the "*Analyze Data*" button, the generation of network XGMM files is started.

Downloading an SSN

After the sequence similarity networks are computed (indicated by an email), datasets containing the full network or rep-node *k* files can be downloaded, see Fig. T3. We recommend using the largest SSN processable by the user's hardware.

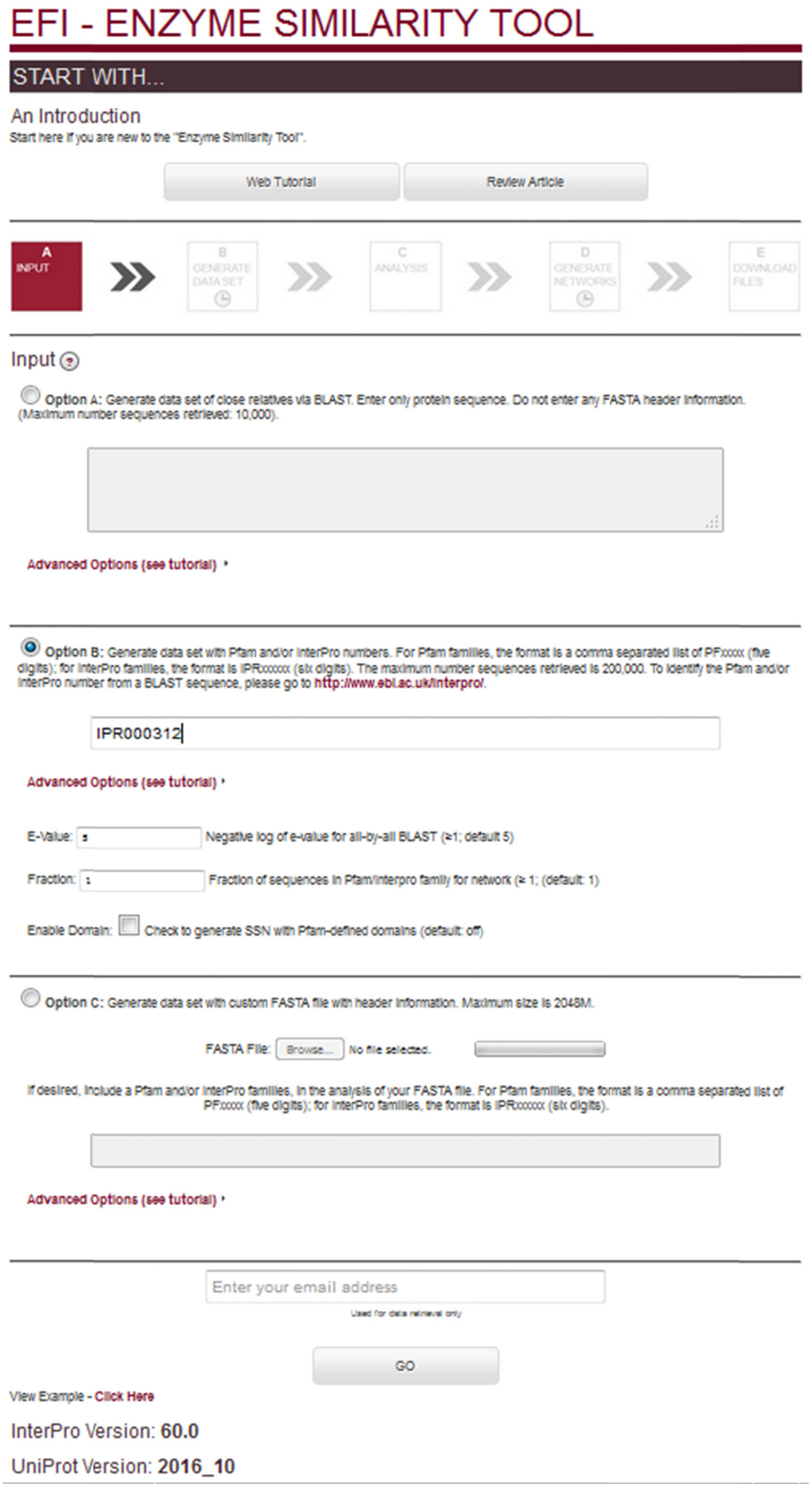

Figure T1: First input mask of the EFI enzyme similarity tool.

The EFI service offers three options to create or enter sequence sets, see http://efi.igb.illinois.edu/efi-est/. Here we used **Option B** and selected the InterPro Family IPR000312. The default parameters for *E-Value, Fraction*, and *Enable Domain* were used.

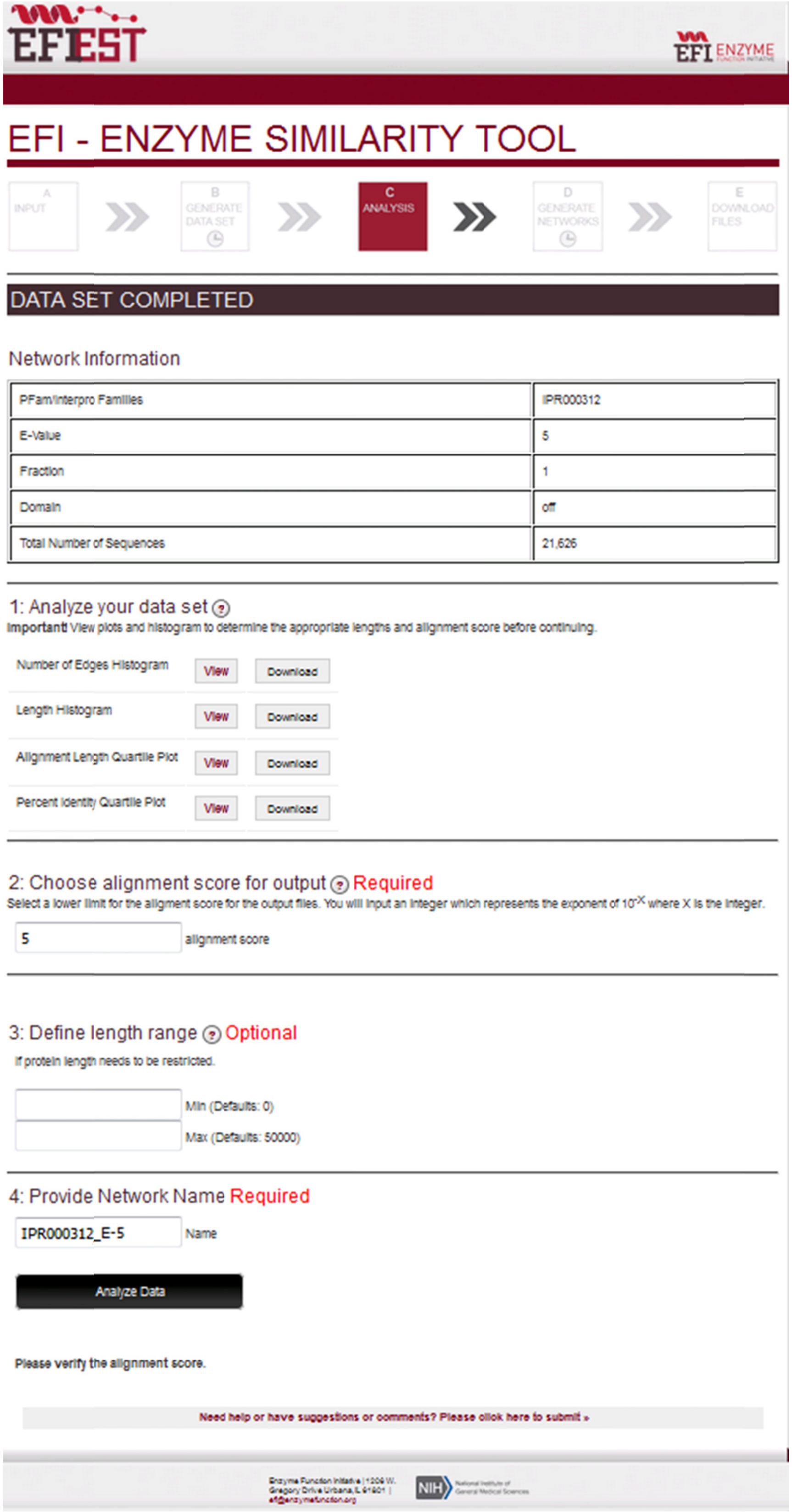

Figure T2: Second input mask of the EFI enzyme similarity tool.

This web page offers statistics of the sequences under study (*Network Information*), histograms showing the distribution of edges and their weight, and plots characterizing the distribution of sequence length. To further process the sequences, a lower limit for the alignment score has to be set and a name for the network has to be provided. Here, the value 5 and the name IPR000312_E-5 were chosen.

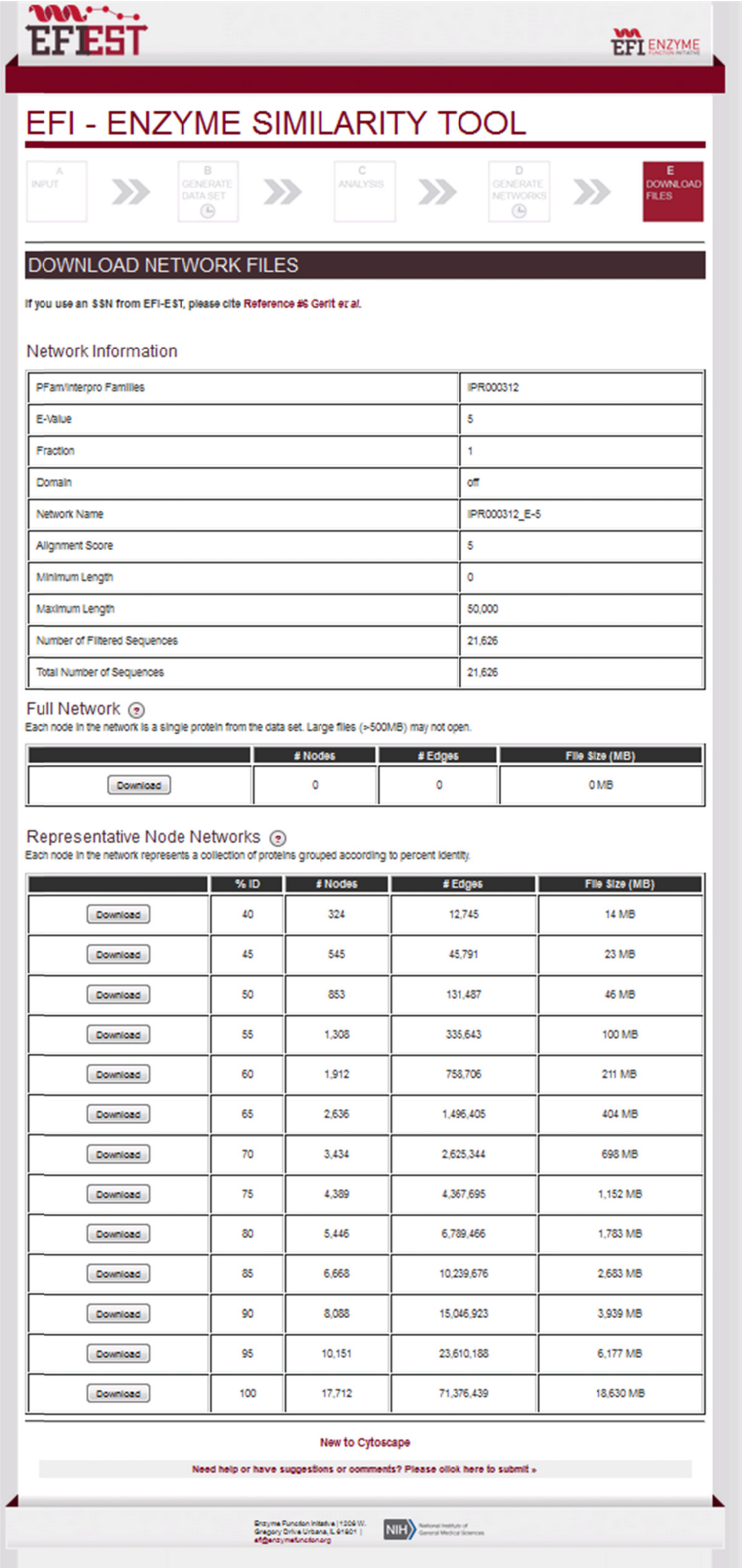
EFIEST

EFI ENZYME FUNCTION INITIATIVE

EFI - ENZYME SIMILARITY TOOL

A INPUT » B GENERATE DATA SET » C ANALYSIS » D GENERATE NETWORKS » E DOWNLOAD FILES

DOWNLOAD NETWORK FILES

If you use an SSN from EFI-EST, please cite Reference #6 Gerlt et al.

Network Information

PFam/Interpro Families	IPR000312
E-Value	5
Fraction	1
Domain	off
Network Name	IPR000312_E-5
Alignment Score	5
Minimum Length	0
Maximum Length	50,000
Number of Filtered Sequences	21,626
Total Number of Sequences	21,626

Full Network

Each node in the network is a single protein from the data set. Large files (>500MB) may not open.

	# Nodes	# Edges	File Size (MB)
Download	0	0	0 MB

Representative Node Networks

Each node in the network represents a collection of proteins grouped according to percent identity.

	% ID	# Nodes	# Edges	File Size (MB)
Download	40	324	12,745	14 MB
Download	45	545	45,791	23 MB
Download	50	853	131,487	46 MB
Download	55	1,308	335,643	100 MB
Download	60	1,912	758,706	211 MB
Download	65	2,636	1,496,405	404 MB
Download	70	3,434	2,625,344	698 MB
Download	75	4,389	4,367,695	1,152 MB
Download	80	5,446	6,789,466	1,783 MB
Download	85	6,668	10,239,676	2,683 MB
Download	90	8,088	15,046,923	3,939 MB
Download	95	10,151	23,610,188	6,177 MB
Download	100	17,712	71,376,439	18,630 MB

New to Cytoscape

Need help or have suggestions or comments? Please click here to submit »

Enzyme Function Initiative | 1206 W. Gregory Drive Urbana, IL 61801 | efi@enzymefunction.org

NIH National Institute of General Medical Sciences

Figure T3: Download page of the EFI SSN tool after the node networks were generated.

The upper part of the html pages represents statistics of the network. The lower part allows one to download networks of different size. For each file, sequences are grouped to nodes according to their similarity, which is determined as % identity (ID). For this tutorial, the rep-node $k = 80$ file resulting from the 80 % ID value was downloaded and renamed to IPR000312_E-5_RN80.xgmml.

Using AGeNNT to create rGNNs

After the download of representative node (rep-node) networks, AGeNNT can be used to compute filtered SSNs and rGNNs. The specific commands for starting AGeNNT can be found in the README.txt file available after installation.

Create a new project: To begin with, the user has to create a *New Project* by clicking the respective button and entering a project name. For the sake of this tutorial, the project IPR000312 is created. After clicking the *OK* button, AGeNNT shows its standard interface.

This interface consists of two windows named *Datasets Window* (shown on the left) and *Results Window* (shown on the right); see Fig. T4. The *Datasets Window* lists in a hierarchical manner all datasets created by subsequent AGeNNT operations. The first line of the *Datasets Window* is the project name, which is here IPR000312. A project may contain several datasets and a dataset is selected for further processing by clicking its name. This activation initiates the update of the *Results Window* and the presentation of context sensitive command buttons displayed below the results. At the moment, the project IPR000312 does not contain a dataset, so we click the "*Add SSN File…*" button.

Add SSN file: By clicking the corresponding button and by choosing the downloaded IPR000312_E-5_RN80.xgmml, AGeNNT reads and analyzes the rep-node file. AGeNNT computes a histogram of edge weights, which is shown in the *Results Window* together with a statistics of the raw network. Using this histogram, AGeNNT determines two alternative thresholds named *A-Th* and *S-Th*, based on heuristics that analyze the shape of the histogram. See the "Methods" section of the manuscript and Fig. T4.

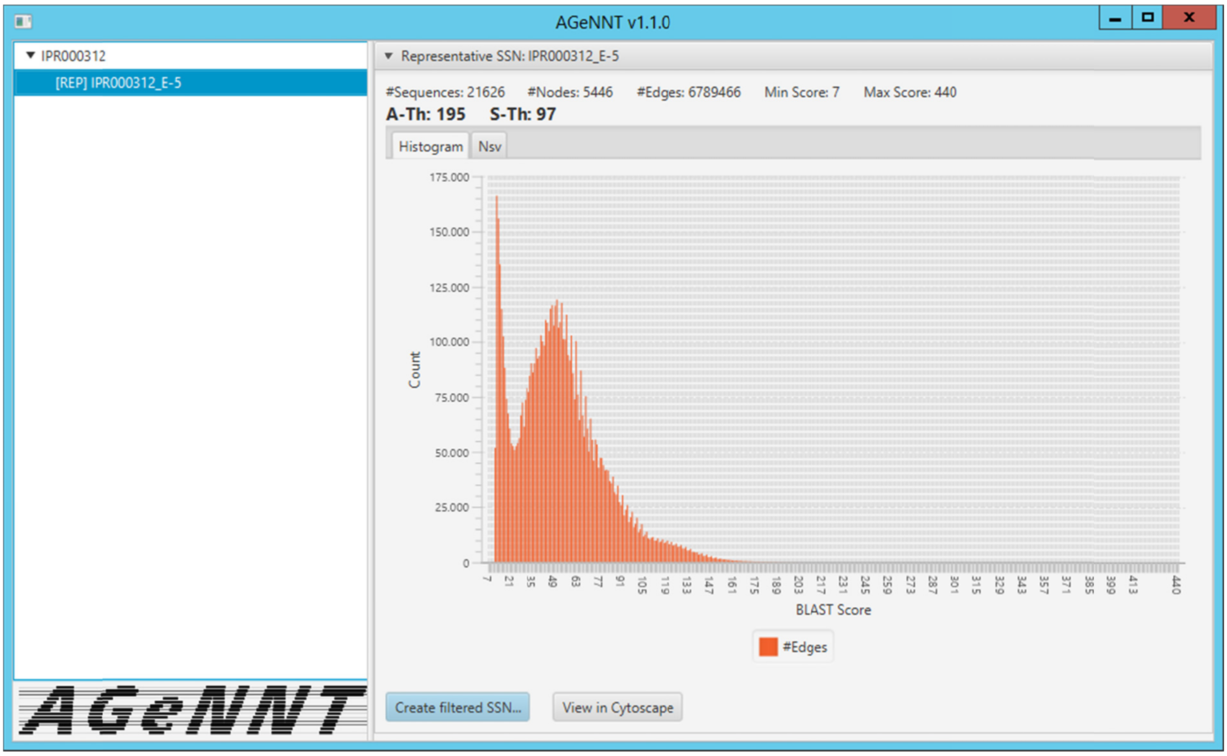

Figure T4: AGeNNT user interface.

It consists of the *Datasets Window* (left) and the *Results Window* (right). The *Datasets Window* lists for each project (named in the first line) in a hierarchical manner the datasets that were created by applying AGeNNT commands. The *Results Window* tabulates the annotation of nodes or displays histograms. Below them, command buttons are arranged in a context sensitive manner. This screenshot shows the edge weight histogram and characteristic features (first line) as well as the values of the two thresholds *A-Th* and *S-Th* for the SSN selected in the *Datasets Window*. After AGeNNT has finished a command, the user has to select (click) the newly generated dataset in the *Datasets Window* for subsequent processing.

Create filtered SSNs: The next step of processing an SSN is to apply a network clustering algorithm. Commonly, only a subset of all SSN edges is utilized, based on a user-defined threshold (*Th*). After having clicked *Create filtered SSN* (Fig. T4), the user has to specify this threshold. Moreover, he has the option to apply a *Taxonomy filter* that eliminates subspecies; see the Methods section. For this tutorial, we used the *S-Th* value (97) and activated the *Taxonomy filter*. By clicking the *OK* button of the corresponding input window, AGeNNT creates the filtered SSN file and after completion, the *Dataset Window* contains a new entry labeled [FIL] > 97 [TAX] indicating the filtering parameters. By clicking this line, AGeNNT updates the histogram of edge scores shown in the *Results Window;* see Fig. T5.

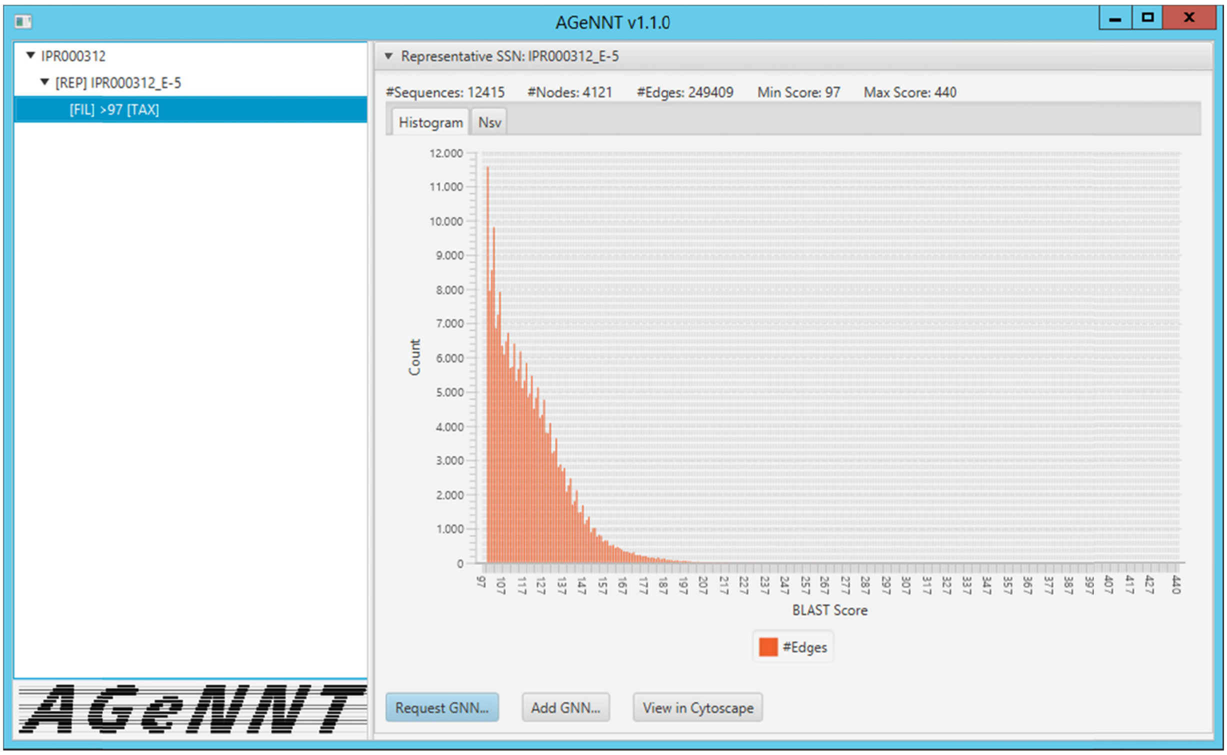

Figure T5: AGeNNT's user interface after the creation of a filtered SSN.

The last entry of the *Datasets Window* indicates that for filtering this rep-node file, a threshold of 97 and the taxonomy filter were applied. The *Results Window* shows the histogram of edge scores resulting from applying these filters.

Create a GNN: After the AGeNNT-specific processing of the SSN, the button *Request GNN* is available; see Fig. T5. Having clicked this command, the user has to specify three parameters required by the corresponding EFI service: Neighborhood size (±3 to ±10 genes), fraction of Pfam co-occurrence within the neighborhoods, and an email address. For this tutorial, a neighborhood of ±10 genes and 20% co-occurrence shall be chosen. Clicking the *Request* button starts the corresponding EFI service.

Note that for large datasets, the computation can take a while. EFI will send the user an email indicating the completion of the job; however AGeNNT downloads the GNN automatically. In cases of a disrupted communication between EFI and AGeNNT, the *Add GNN* button can be used to retrieve the dataset after the user has received the email indicating the termination of the EFI service. The user simply has to copy/paste the respective URL from the email to the AGeNNT command window.

Here, AGeNNT has loaded the GNN automatically and the new dataset ([GNN] nh=10 co=20) is represented by a table listed in the *Results Window*; see Fig. T6.

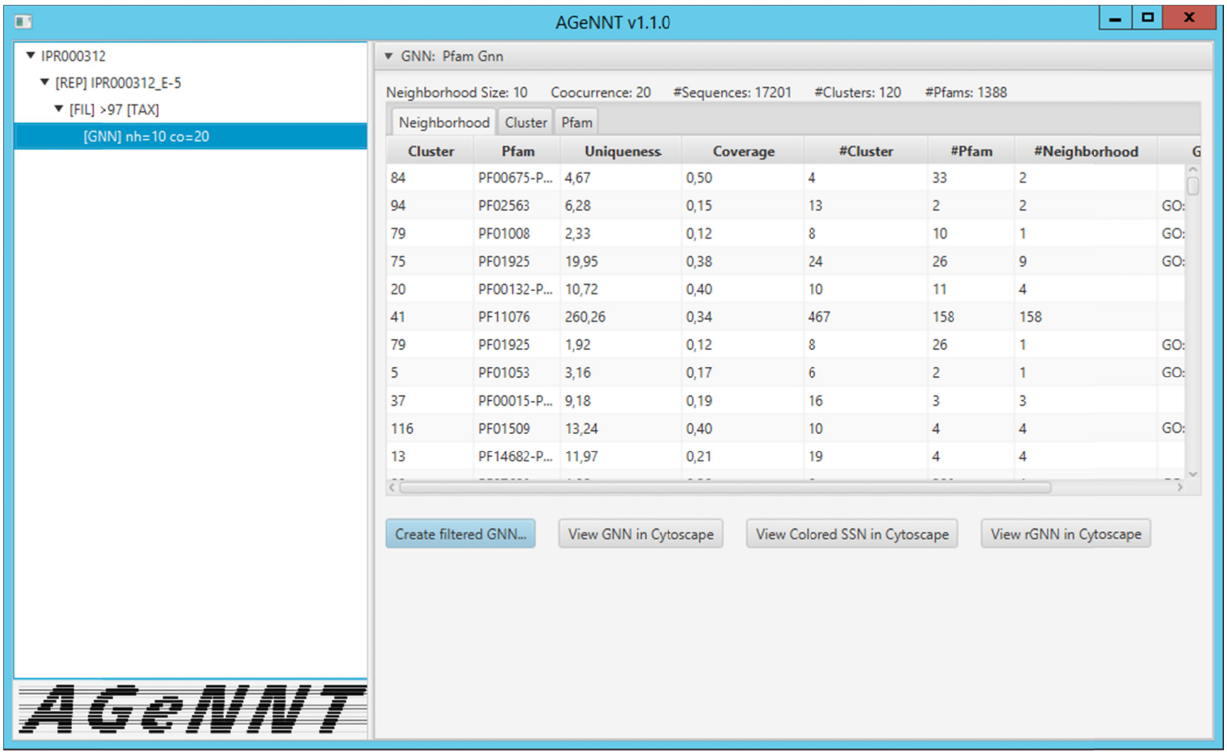

Figure T6: AGeNNT user interface after the creation of a filtered SSN.

This (raw) GNN was created by the EFI service and contains the Pfam-nodes that were found in all GNs by scanning a neighborhood of ±10 with a co-occurrence of 20%.

Create filtered GNN: The (raw) GNN compiled by the EFI service contains Pfam-nodes describing the function of all GNs. The user can initiate the elimination of protein functions he is not interested in, like transporters or transcription factors. Clicking the *Create filtered GNN* button allows him to choose the built-in whitelist or his own one; see Methods. Having applied AGeNNT's filter and chosen the newly computed dataset, AGeNNT offers to display three datasets by means of Cytoscape: The corresponding buttons are labeled *View GNN in Cytoscape*, *View Colored SSN in Cytoscape* and *View rGNN in Cytoscape;* see Fig. T7. For the latter two datasets, cluster-nodes are colored with the same coloring scheme.

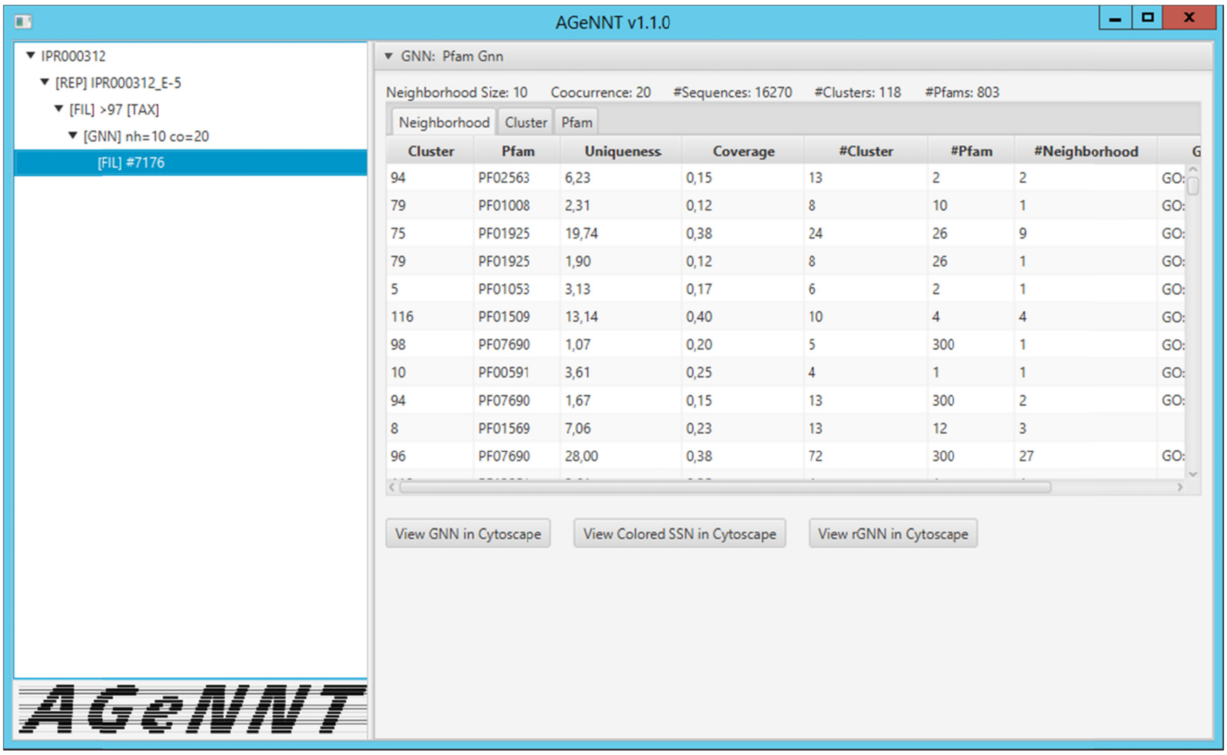

Figure T7: AGeNNT user interface after the application of a whitelist.

The existence of a new dataset ([FIL]#7176) shown in the *Datasets Window* indicates that the whitelist was applied.

Using Cytoscape to display and analyze rGNNs

After the application of AGeNNT's filter routines, all datasets are ready to be visualized and further processed by means of Cytoscape. For a seamless analysis, AGeNNT allows the user to start corresponding program sessions by clicking buttons.

View an SSN: Clicking the *View Colored SSN in Cytoscape* button starts Cytoscape to visualize the corresponding SNN. Commonly, the *organic layout* is well suited to visualize an SSN (Cytoscape drop-down menu "*Layout > y-files > organic y-files layout*". The EFI-EST service chooses different colors and symbols to distinguish elements belonging to different sequence clusters. AGeNNT uses the same scheme for the labelling of the rGNN cluster-nodes which helps to identify corresponding entries.

View an rGNN: Clicking the *View rGNN in Cytoscape* button starts a Cytoscape session allowing for the visualization of the rGNN. Again, the *organic layout* is well suited for a first representation. Fig. T8 shows the organization of the Cytoscape interface. In addition to the *Menu* and *Tool Bars* arranged on top, it consists of three panels: These are the *Network View Panel*, which displays the selected network, the *Table Panel* which displays properties of selected nodes or edges, and the *Control Panel*. The latter lists the network under study (Panel *Network*), the way it is represented (Panel *Style*), and filters to select certain network elements (Panel *Select*).

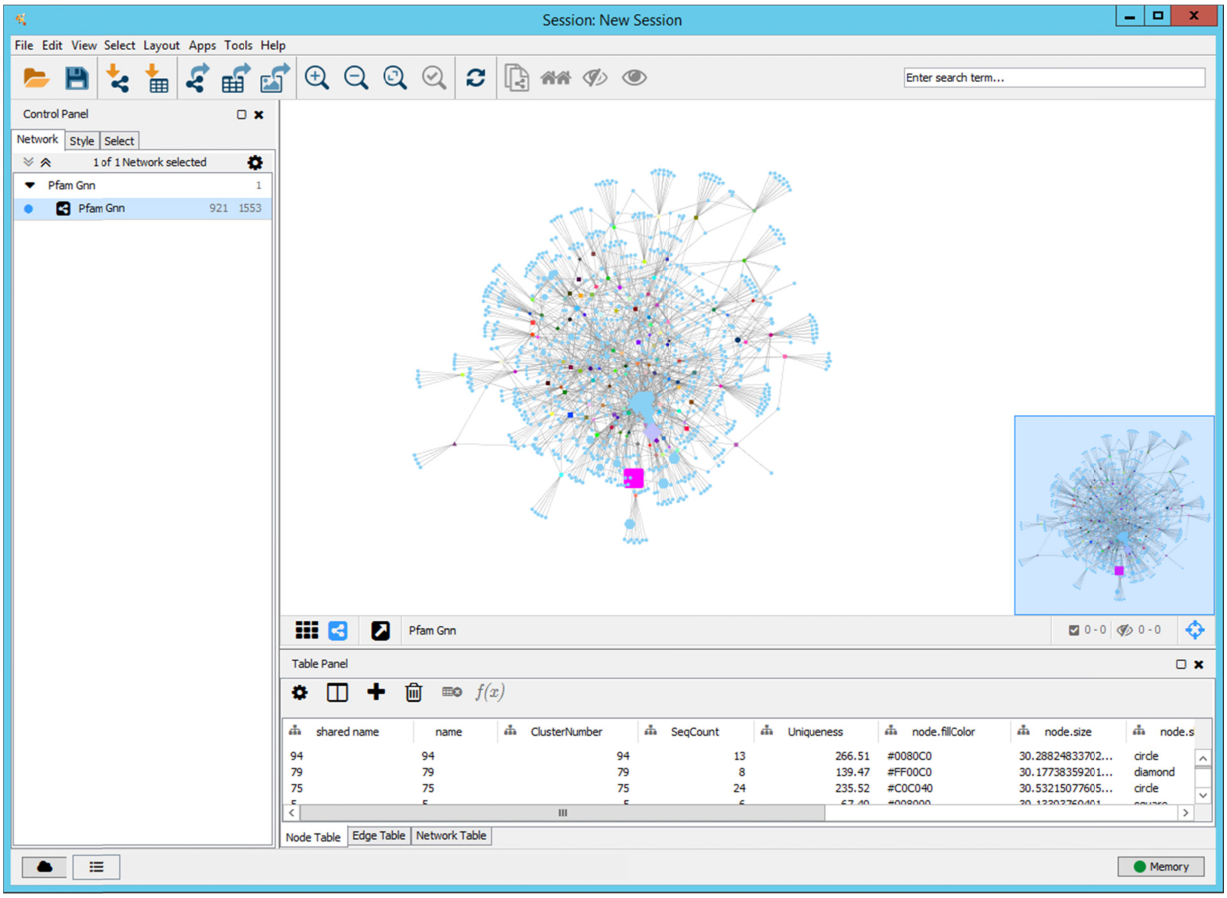

Figure T8: Initital view of an rGNN.

The rGNN shown in the *Network View Panel* (top right) is dominated by many specific GNs. To eliminate them, the user can apply a *Node:SeqCount* filter and hide or delete all nodes representing a small number of sequences. The filter is implemented in the *Control Panel(Select)* (left). The commands for hiding or deleting nodes and edges are part of the *Select* command to be found in the *Menu Bar* (top).

Simplifying the rGNN by applying Cytoscape commands: Fig. T8 makes clear that the initial representation of this rGNN is overly complex due to many highly specific GNs. In order to eliminate less abundant GNs, the user can utilize the Cytoscape *Control Panel(Select)*, apply a *Node:SeqCount* filter and hide or delete all nodes with a small number of sequences. Moreover, the user can interactively rearrange the position of nodes in order to group cluster- and/or Pfam-nodes. Fig. T9 shows the network after the filter *Node:SeqCount is between 1 and 150 inclusive* and the *Hide selected nodes and edges* command were applied. Subsequently, the *organic layout* is utilized followed by a minor rearrangement of nodes.

Determine the enzyme functions found in a specific GN: In order to select the Pfam-nodes constituting a specific GN, it is sufficient to first click a cluster-node and then to use the shortcut <Ctrl>6. This Cytoscape command selects all nodes interconnected to a cluster-node and their annotation is then listed in the *Node Table* of Cytoscape's *Table Panel*; compare Fig. T9.

Controlling uniqueness values, the phylogenetic distribution, GO terms and other properties of nodes and edges: All properties of nodes and edges are listed in the *Table Panel* which is subdivided in a *Node Table* and an *Edge Table*. After having clicked nodes or edges of interest, the panels list corresponding features.

Changing display attributes: Color, size, and shape of nodes and edges can be altered by means of the *Control Panel(Style)*.

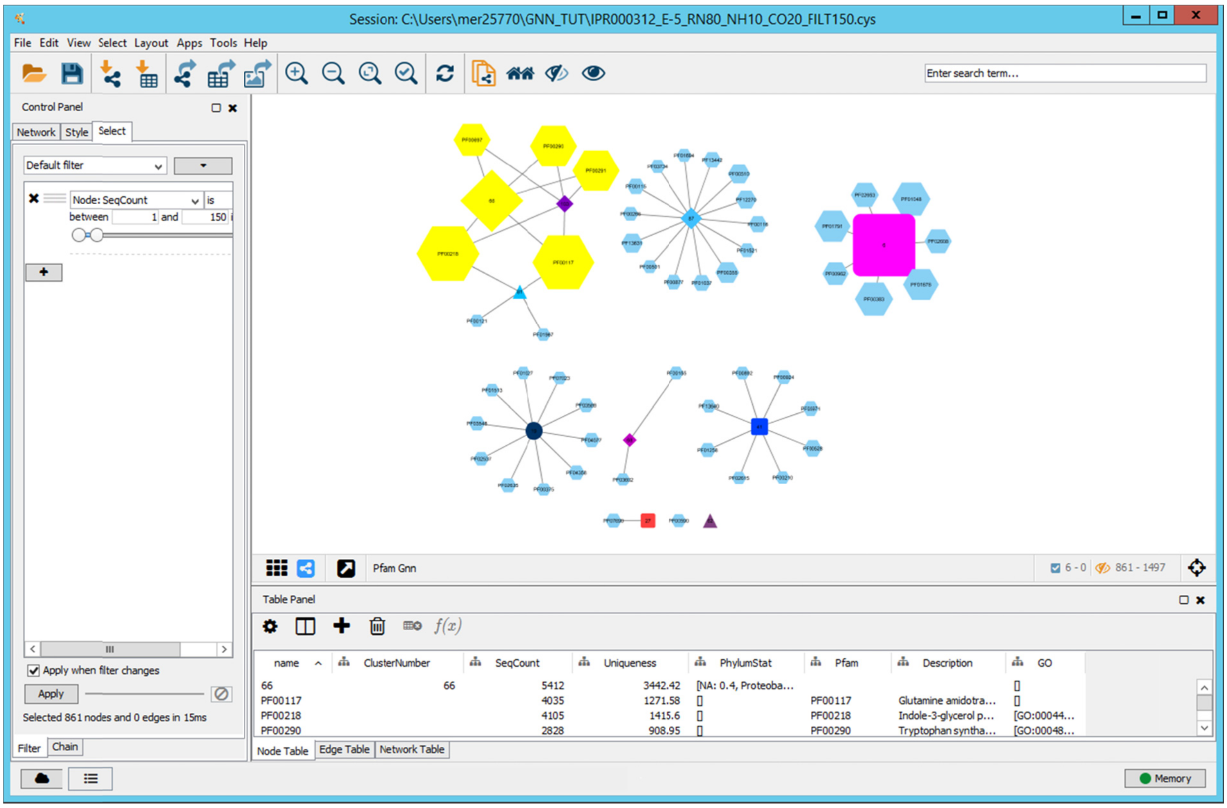

Figure T9: Listing the enzyme functions encoded in the GN of cluster-node 66.

In order to study the function of enzymes constituting the GN of cluster-node 66, this node was clicked. The shortcut <Ctrl>6 selects all adjacent Pfam-nodes (highlighted in yellow), and their annotation is shown in the *Table Panel* below the graph. The annotation offered by AGeNNT contains also uniqueness values and a statistic indicating the phylogenetic origin of the species contributing to the cluster-node. For the Pfam-nodes, their Pfam-ID, description of the function and GO terms are shown. Individual edges are further characterized by their coverage.
