# Supplementary material for: AGeNNT: annotation of enzyme families by means of refined neighborhood networks
Source: BMC Bioinformatics. 2017 May 25;18:274. doi: 10.1186/s12859-017-1689-6 (PMC5445326; doi:10.1186/s12859-017-1689-6)
Supplement: Supplementary file 2 — Representations of 27 rGNNs resulting from a systematic variation of parameters used to generate them and of rGNN_7115_30. (PDF 280 kb) [file 12859_2017_1689_MOESM2_ESM.pdf]

AGeNNT: annotation of enzyme families by means of refined neighborhood networks

Florian Kandlinger[1,2], Maximilian G. Plach[1] and Rainer Merkl[1*]

[1]Institute of Biophysics and Physical Biochemistry, University of Regensburg,
D-93040 Regensburg, Germany

[2]Faculty of Mathematics and Computer Science, University of Hagen,
D-58084 Hagen, Germany

Supplementary Figures S1 – S4

Figure S1: Nine rGNNs determined for the rep-node 100 file of IPR000312.

For the generation of these rGNNs, the three values ±10, ±6, and ±3 were chosen for the neighborhood size *nb* and the values 10%, 20%, and 30% were chosen for the co-occurrence to test all nine combinations of these two parameters. For visualization, Cytoscape was used and all nodes representing < 150 sequences were eliminated analogously to the generation of Fig. 1. For all representations of cluster-nodes, the same color code was used. Cytoscape's organic layout was applied initially, and the position of nodes was rearranged interactively for ease of interpretation.

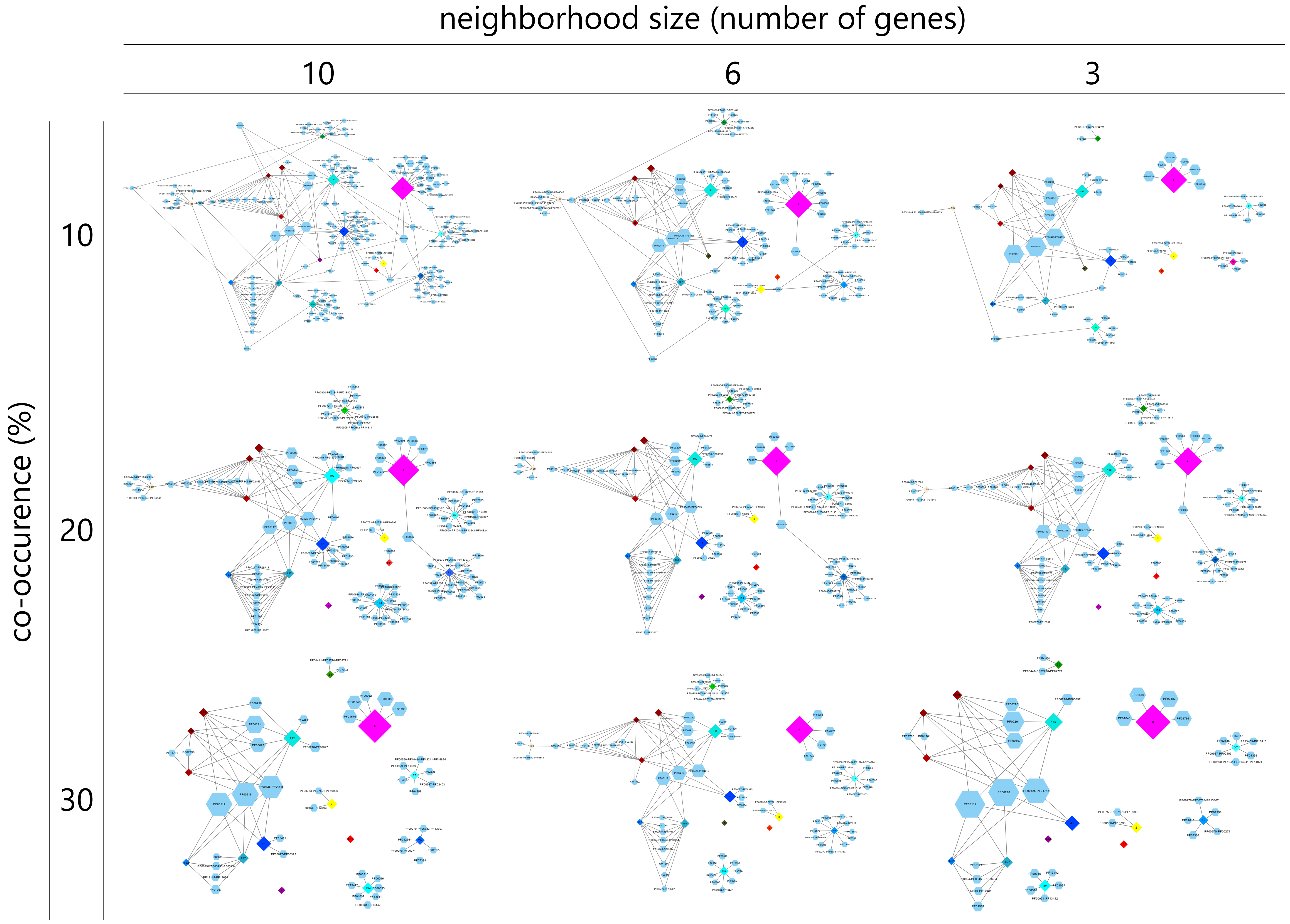

Figure S2: Nine rGNNs determined for the rep-node 80 file of IPR000312.

For the generation of these rGNNs, the three values ±10, ±6, and ±3 were chosen for the neighborhood size *nb* and the values 10%, 20%, and 30% were chosen for the co-occurrence to test all nine combinations of these two parameters. For visualization, Cytoscape was used and all nodes representing < 150 sequences were eliminated analogously to the generation of Fig. 1. For all representations of cluster-nodes, the same color code was used. Cytoscape’s organic layout was applied initially, and the position of nodes was rearranged interactively for ease of interpretation.

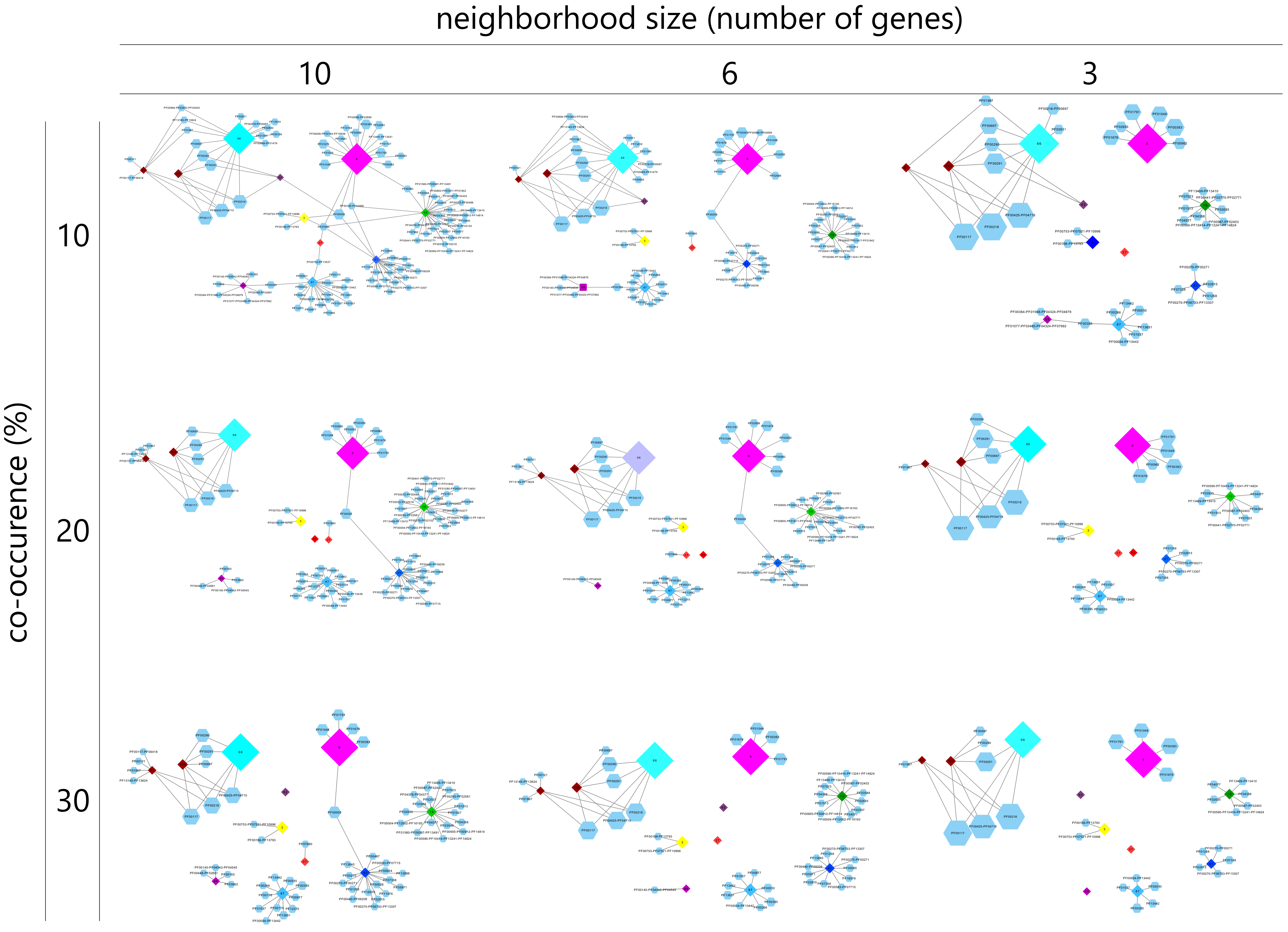

Figure S3: Nine rGNNs determined for the rep-node 60 file of IPR000312.

For the generation of these rGNNs, the three values ±10, ±6, and ±3 were chosen for the neighborhood size *nb* and the values 10%, 20%, and 30% were chosen for the co-occurrence to test all nine combinations of these two parameters. For visualization, Cytoscape was used and all nodes representing < 150 sequences were eliminated analogously to the generation of Fig. 1. For all representations of cluster-nodes, the same color code was used. Cytoscape's organic layout was applied initially, and the position of nodes was rearranged interactively for ease of interpretation.

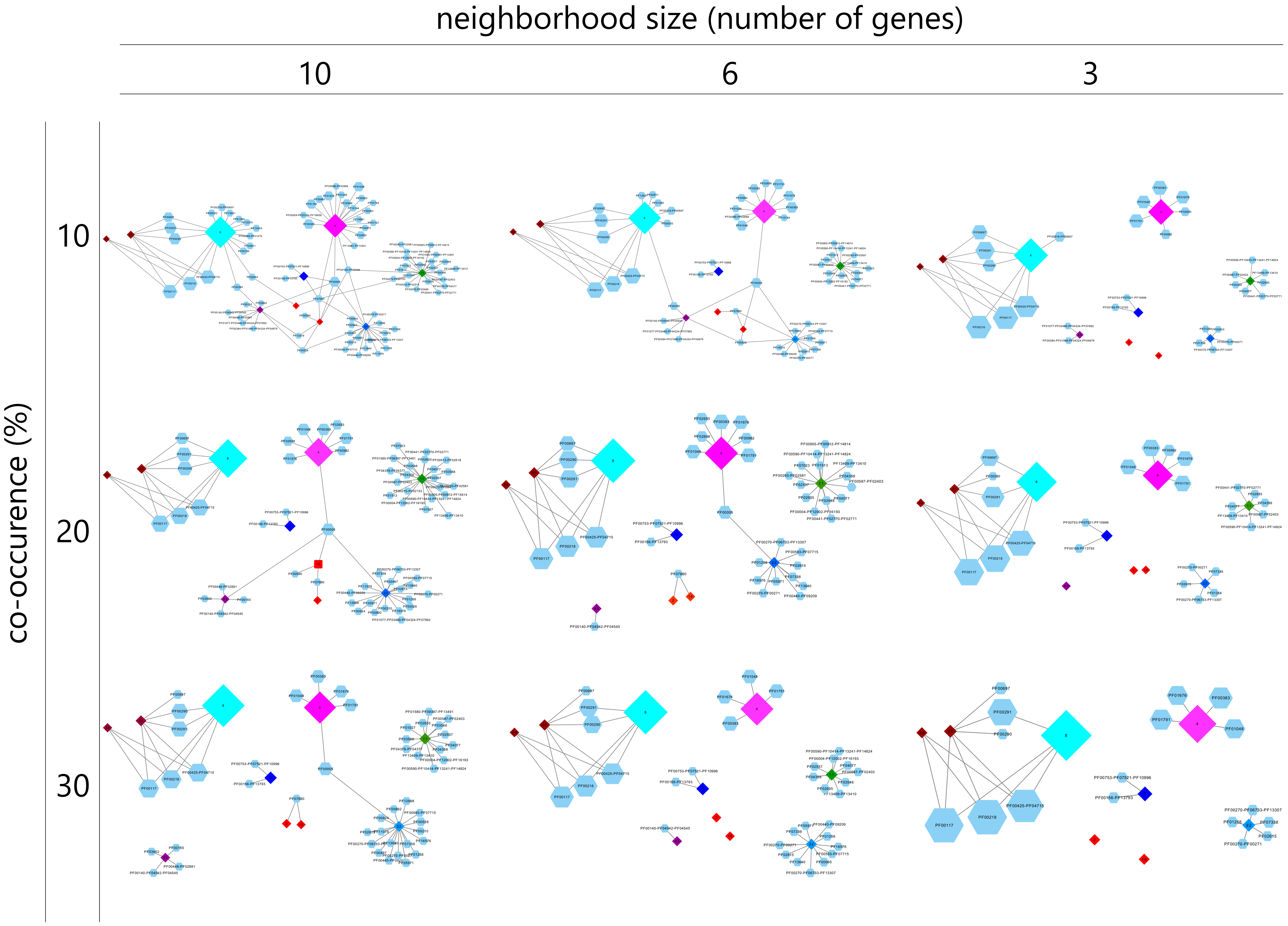

Legend of Figure S4: rGNN (named rGNN_7115_ 30) determined for IPR007115.

In agreement with the rGNN shown in Fig. 5, this rGNN is based on the rep-node 80 file and was generated with a neighborhood size of 10 and a co-occurrence of 20%. In contrast to Fig. 5, only those nodes were eliminated that represented not more than 30 sequences. The motifs that are specific for the different PTPS functionalities and findings related to the co-localization of genes were taken from [1, 2]. Sequence motifs were determined for each cluster-node *k* by opening the corresponding SSN and by selecting the UniProt identifiers related to sequence cluster *k*. After the elimination of special characters like "[]", the UniProt web-service *Retrieve/ID mapping* (http://www.uniprot.org) was used to download the sequences and an MSA was generated by means of MAFFT [3]. MSAs were analyzed by means of Aliview [4].

The central region of the rGNN contains the PTPS-I homologs, which are involved in queosine biosynthesis. Their common genomic neighborhood is characterized by the Pfams PF04055-PF13394 (radical SAM superfamily-4Fe-4S single cluster domain; includes queosine biosynthesis enzyme QueE), PF06508 (Queuosine biosynthesis protein QueC), and PF14489 (QueF-like protein). The corresponding cluster-nodes (65, 15, 47, 8, 29, 91, 23, 63, and 33) represent a total of 4065 sequences of the InterPro family. Thus, PTPS-I function is the most prevalent functionality of IPR0071115. Sequences from Firmicutes dominate cluster-nodes 91 and 23; this abundance explains differences in corresponding GN composition: For example, QueE homologs comprise a different domain architecture, which contains PF04055-PF13394 in Firmicutes and PF04055-PF13353 in the other species. In comparison to rGNN_7115_ 150 (Fig. 5) this rGNN contains many smaller PTPS-I clusters and a further, QueC-like enzyme. Importantly, all bona fide PTPS-I cluster-nodes exclusively represent sequences that contain the PTPS-I typical CxxxHGH sequence motif.

A further, distinct subnet is constituted by the cluster-nodes on the top right (71, 5, 56). The GNs of these three cluster-nodes contain PF01227 (GTP cyclohydrolase I; includes the folate biosynthetic enzyme FolE) and this co-localization as well as the co-localization of PF00106 (short-chain dehydrogenase; includes sepiapterin reductase (SR)) determined for cluster-node 56 are a property of PTPS-II homologs. Additionally, all the sequences in cluster-node 71 contain the PTPS-II typical sequence motif CxxxxxHGH. The sequences in cluster-nodes 5 and 56 contain the motifs CxxxxHGH and CxxxHGY, thus we termed them PTPS-II-like. Interestingly, the GN of cluster-node 29 (most likely PTPS-I functionality) also contains PF01227 (FolE). The PhylumStat distribution shows that this cluster-node contains many Clostridial sequences, which co-localize for unclear reasons with the folE gene.

Cluster-nodes 62 and 55 form a further, distinct subnet and the corresponding sequences contain a PTPS-IV-typical motif. Their corresponding GNs contain PF00925 (GTP cyclohydrolase I; includes the folate biosynthetic enzyme FolE2) and PF01872 (RibD C-terminal domain). RibD is involved in riboflavin biosynthesis, which seems also to be a characteristic of PTPS-IV homologs [1, 2]. Importantly, our rGNN approach identified FolE2 as an element of the PTPS-IV neighborhood, which has not been recognized previously.

The sequences of cluster-node 82 share the PTPS-III-specific motif (ExxHGH). However, the GN that contains PF13394 (4Fe-4S single cluster domain) and PF00293 (Nudix family) does not shed much light on their function. Nudix enzymes are phosphohydrolases that hydrolyze a wide range of organic pyrophosphates, including nucleoside di- and triphosphates, dinucleoside and diphosphoinositol polyphosphates, nucleotide sugars, and RNA caps, with varying degrees of substrate specificity. The dominating cluster-node 71 represents PTPS-II functionality; the GN seems only marginally conserved and contains only PF01227 (FolE) and PF00293 (Nudix family). Their low coverage values might at least partially be due to eukaryotic sequences contributing to cluster-node 71. Cluster-node 5 may represent a further PTPS functionality, the GN contains PF01142 (tRNA pseudouridine synthase D) and PF03167 (DNA glycosylases).

Figure S4: rGNN (named rGNN_7115_ 30) determined for IPR007115.

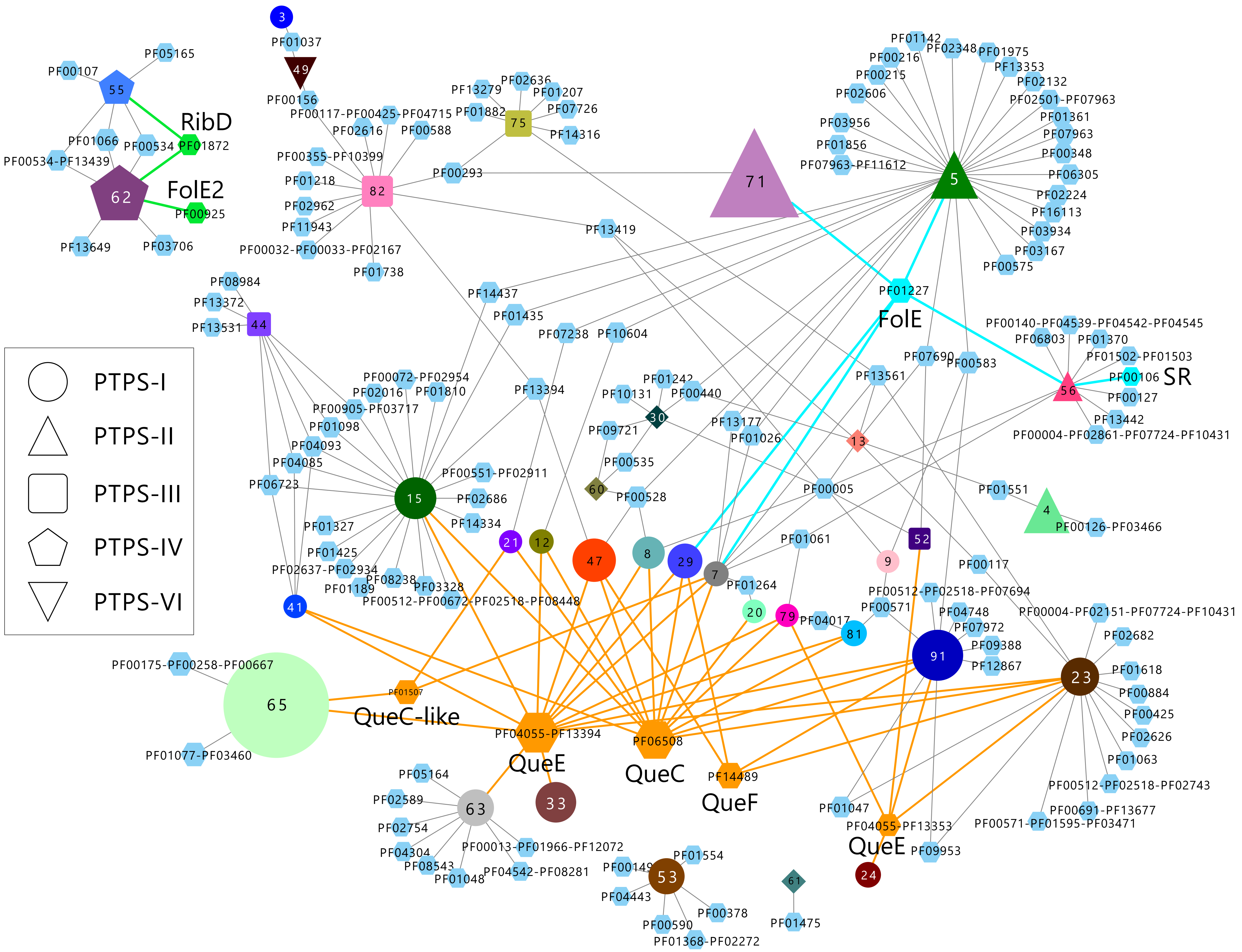

Supplementary References

[1] Zallot R, Harrison KJ, Kolaczkowski B, de Crécy-Lagard V, Functional annotations of paralogs: a blessing and a curse. Life (Basel). 2016; 6:

[2] Phillips G, Grochowski LL, Bonnett S, Xu H, Bailly M, Blaby-Haas C, El Yacoubi B, Iwata-Reuyl D, White RH, de Crécy-Lagard V, Functional promiscuity of the COG0720 family. ACS Chem Biol. 2012; 7:197-209.

[3] Katoh K, Standley DM, MAFFT multiple sequence alignment software version 7: improvements in performance and usability. Mol Biol Evol. 2013; 30:772-780.

[4] Larsson A, AliView: a fast and lightweight alignment viewer and editor for large datasets. Bioinformatics. 2014; 30:3276-3278.
